# A Derivatization-Free GC–MS Method for Reliable Quantification of Short-Chain Fatty Acids in *Sparus aurata*

**DOI:** 10.3390/metabo16090636

**Published:** 2026-08-31

**Authors:** Violeta Kalemi, Luca Chiodaroli, Simona Rimoldi, Genciana Terova

**Affiliations:** Department of Biotechnology and Life Sciences, University of Insubria, Via J.H. Dunant, 3, 21100 Varese, Italy; vkalemi@uninsubria.it (V.K.); luca.chiodaroli.83@gmail.com (L.C.); simona.rimoldi@uninsubria.it (S.R.)

**Keywords:** acetate, butyrate, GC-MS chromatography, extraction, gilthead sea bream, short-chain fatty acid, propionate, fish

## Abstract

**Highlights:**

**What are the main findings?**

- The optimized GC–MS method showed high linearity, sensitivity, and minimal matrix effects in fish fecal samples.
- Salt addition significantly improves phase separation between the aqueous and organic solvent fractions during extraction.

**What are the implications of the main findings?**

- The method enables direct quantification of acetate, propionate, and butyrate in fish feces without derivatization.
- This approach supports reliable profiling of fecal volatile fatty acids for applications in fish nutrition, gut microbiota, and metabolomics studies.

**Abstract:**

**Background/Objectives:** This study presents a gas chromatography–mass spectrometry (GC–MS) method for the derivatization-free quantification of the three major short-chain fatty acids (SCFAs)—acetate, propionate, and butyrate—in gilthead sea bream (*Sparus aurata*) fecal samples. SCFAs are key metabolites involved in intestinal physiology and are widely recognized as indicators of gut microbiota activity and host health across animal species. In aquaculture research, SCFA profiling can provide valuable insight into diet–microbiota interactions and gut functional status. **Methods:** The method was developed and optimized using gut-content samples collected from 105 gilthead sea bream at the end of two independent feeding trials, yielding 42 pooled samples (18 from FT1 and 24 from FT2). An extraction protocol based on acidified aqueous extraction with water followed by liquid–liquid extraction using methyl tert-butyl ether was developed (ExA) and subsequently optimized (ExB) to improve extraction consistency and efficiency. SCFA separation and quantification were performed by GC–MS using external calibration. Method validation included evaluation of linearity, matrix effects, and partition coefficients between water and methyl tert-butyl ether. **Results:** The optimized extraction method (ExB) demonstrated high analytical performance, with linearity coefficients exceeding 0.992 and matrix effects below 17.4%. **Conclusions:** The proposed method represents a practical and reliable approach for routine SCFA analysis in fish fecal samples. It provides a useful analytical tool for studies investigating diet–microbiota interactions, gut health, and functional responses to nutritional interventions in aquaculture species.

## 1. Introduction

Short-chain fatty acids (SCFAs) are major microbial metabolites that play important roles in host physiology, energy homeostasis, and immune regulation. Acetate, propionate, and butyrate are the predominant SCFAs produced through microbial fermentation of dietary fibers and have been associated with gut health in both mammals and fish, including aquaculture species [1,2,3,4]. Consequently, reliable SCFA quantification is essential for investigating host–microbiome interactions and nutritional interventions in fish. However, the analysis of SCFAs in fish fecal matter remains analytically challenging because of the limited amount of biological material available, the low concentrations of these metabolites, and the complexity of the fecal matrix. These factors require extraction procedures that maximize recovery while minimizing matrix effects, yet only a limited number of methods have been specifically developed and validated for fish fecal samples. Moreover, SCFAs act as key metabolic intermediates influencing nutrient utilization, lipid deposition, and energy balance, ultimately affecting the composition and quality of edible tissues in food-producing animals.

The analytical assessment of SCFAs in fish fecal matter poses a particular challenge because the presence of thin mucus layers, and the rapid epithelial absorption of SCFAs lead to heterogeneous luminal distributions and make it difficult to determine concentrations representative of actual microbial production [5]. Gas chromatography–mass spectrometry (GC-MS) is widely regarded as a reliable analytic approach due to its high repeatability, extensive compound identification libraries and high sensitivity to relatively low concentrations of analytes. Other analytical approaches are also available for quantifying SCFAs, notably LC–MS or LC–MS/MS [1,6,7,8,9] and HPLC–UV [10,11,12]. SCFA profiling has also been applied in nutritional studies in gilthead sea bream using alternative analytical approaches [3].

GC-MS enables either direct analysis or chemical derivatization to enhance analyte volatility and sensitivity [2,13]. Derivatization methods, such as labeling with aniline or 3-nitrophenylhydrazine, can improve chromatographic resolution and lower detection limits [1]. Consequently, many analytical methods for SCFA quantification rely on derivatization. However, derivatization may also introduce quantification artifacts resulting from incomplete reactions, differences in derivatization efficiency between standards and samples, matrix effects, and increased procedural complexity [14]. For these reasons, derivatization-free approaches have attracted increasing interest because they reduce experimental bias, simplify sample preparation, and preserve SCFAs in their native form [13,14,15].

The development of a GC-MS method tailored for small quantities of fish fecal matter is important for comparative studies. Also, highly sensitive and highly repeatable methods are particularly necessary when working with fish faecal samples, which are typically available in limited amounts and may contain SCFAs at much lower concentrations than mammalian fecal matter. Recent advances demonstrate that extraction and enrichment strategies, including solvent-based extraction and solid-phase purification, can achieve high recovery and low detection limits [13,14,15], making them well suited for GC-MS analysis.

To address these challenges, we adapted a protocol originally developed by Yao et al. [16] for human serum, in which SCFAs are extracted following acidification with methyl tert-butyl ether (MTBE). This protocol served as the foundation for method development, as the low viscosity of fish fecal suspension and the low sample mass more closely resemble serum than the complex, highly viscous intestinal contents and heterogeneous matrix of mammalian fecal matter. Furthermore, the extraction procedure, along with sample and reagent quantities, could be readily adapted to accommodate fish fecal samples.

The primary objective of this study was to develop a derivatization-free GC–MS method for the quantification of three major short-chain fatty acids (acetic, propionic, and butyric acids) in low-mass fish fecal samples. These metabolites are the predominant products of intestinal microbial fermentation and are directly associated with key functional bacterial groups in the gut. Findings from Czarnowski et al. [2], Kim et al. [13], and Zheng et al. [15] were integrated to optimize quantification strategies, evaluate matrix effects, and compare the sensitivity of different SCFA measurement approaches. Accordingly, this study presents a modified extraction-based method for SCFA quantification in fish feces. By adapting and refining serum-based protocols, we established a derivatization-free workflow design to minimize analytical bias, reduce artifacts, ensure high sensitivity, and enable accurate SCFA profiling in fish gut.

## 2. Materials and Methods

### 2.1. Instruments and Solutions

The reagents and analytical standards used for sample preparation and GC-MS analysis are described in this section.

The reference standards and reagents, including butyric acid (purity ≥ 99.5%), propionic acid (purity ≥ 99.5%), acetic acid (purity ≥ 99.0%), methyl tert-butyl ether (MTBE, purity 99.9%), sodium sulphate and magnesium chloride, were purchased from Sigma-Aldrich (Milan, Italy). Hydrochloric acid 37% was obtained from Carlo Erba Reagents (Milan, Italy). Water was purified with the Millipore Elix 5 system (Burlington, MA, USA).

Standard dilutions were prepared on ice and stored at −80 °C for long-term storage. Samples could also be stored at −20 °C for several months.

Two reference compounds with complementary and clearly distinct functions were used to independently monitor extraction-related and injection/instrumental variability, thereby allowing these two sources of analytical variation to be evaluated separately. Biphenyl was used as a recovery/surrogate standard to monitor the consistency of MTBE phase separation and organic-phase recovery. Owing to its low solubility in water and high affinity for organic solvents, biphenyl provides an indicator of the behavior and recovery of the MTBE phase; however, because its physicochemical properties and partitioning behavior differ substantially from those of SCFAs, it was not used to correct analyte-specific SCFA extraction recovery. A 10 µg/mL solution of biphenyl in MTBE (MTBE-BPHE) was prepared from biphenyl crystals, stored at −20 °C, and used as the extraction solvent. Ethyl butyrate (ethyl butanoate) was used as an internal standard to monitor injection consistency and instrumental variability because of its chemical similarity to the target compounds and its distinct retention time [9]. A 0.01 mM solution of ethyl butyrate in MTBE (MTBE-EB) was prepared and used to dilute sample extracts prior to GC–MS analysis. This complementary use of biphenyl and ethyl butyrate enabled extraction-related variability to be distinguished from injection- and instrument-related variability during method optimization.

Samples were analyzed using a Trace™ 1600 series gas chromatograph (Thermo Fisher Scientific, Austin, TX, USA) coupled with an ISQ™ 7610 single quadrupole mass spectrometer (Thermo Fisher Scientific, Austin, TX, USA).

The GC–MS system was equipped with a TraceGOLD™ TG-5MS column (30 m × 0.25 mm × 0.25 μm) (Thermo Fisher Scientific, Waltham, MA, USA) or a Nukol™ capillary GC column (30 m × 0.25 mm × 0.25 μm) (Supelco, Bellefonte, PA, USA).

### 2.2. Sample Acquisition

The gut contents were collected from a total of 105 gilthead seabream (*Sparus aurata*) at the end of two independent feeding trials (FT1 and FT2). The two trials were conducted under comparable husbandry conditions and employed the same dietary treatments, although fish differed in size. In both trials, fish were allocated to three dietary groups and received either a fish-oil-based control diet or one of two experimental diets in which fish oil was partially replaced with a microalgal oil included at 0.4% or 2%. The two feeding trials were conducted independently and were used as sources of biological matrices for analytical method development rather than for direct between-trial biological comparison. At the end of the feeding trials, the feeding rate corresponded to 1.5% and 2.2% of fish biomass in FT1 and FT2, respectively. The day prior to sampling, fish were fed to apparent satiation in the afternoon to ensure adequate feed digestion and sufficient intestinal content on the day of sampling. Fecal matter from the entire length of the intestine were collected in 5 mL centrifuge tubes. When necessary, fecal material from 2 to 3 fish was pooled to obtain sufficient material for the extraction protocol. As a result, 42 samples were obtained for further processing (FT1, 18 samples, FT2, 24 samples). Each sample weighed approximately 1 g. The material was stirred briefly with a metallic spatula to ensure homogeneous mixing of fecal material originating from different fish. Samples were stored on dry ice at the sampling site and at −80 °C until processing, to prevent degradation and volatilization of fatty acids.

### 2.3. Sample Preparation

Samples were processed within 7 to 10 days after sampling. Fecal samples were partially thawed on ice to facilitate aliquoting of the required amount for extraction, while the remaining material was stored at −80 °C for future use. Samples were maintained on ice throughout the extraction procedure, and all centrifugation steps were performed at 4 °C. Extracts were stored at −80 °C in 1.5 mL microcentrifuge tubes sealed with Parafilm^®^ M (Amcor, Neenah, WI, USA) and analyzed by GC-MS as soon as possible.

### 2.4. Extraction of SCFAs from Fecal Samples

**ExA—first method adaptation:** This method was adapted from the protocol described by Yao et al. [16], selected because of the similar physical characteristics of the investigated samples and its suitability for SCFA quantification. ExA was applied to 18 pooled samples from trial FT1.

A 1 g aliquot of partially thawed fecal material was extracted with 10 mL of 1.2 M aqueous hydrochloric acid (HCl) by vortexing for 10 min. The aqueous phase was separated by centrifugation at 3500× *g* and 4 °C for 10 min. The recovered aqueous solution was extracted with 1 mL MTBE-BPHE and vortexed for 2 min. The organic phase was recovered by centrifugation at 3500× *g* and 4 °C for 5 min. Three additional serial extractions were subsequently performed using 200 µL of fresh MTBE. The organic phase obtained from each extraction step were stored separately for GC-MS analysis.

**ExB—second method adaptation:** This method was developed as a modification of ExA based on practical limitations encountered during its application to the first set of samples (FT1). In particular, variable MTBE recovery during matrix-matched calibration resulted in inconsistent extract volumes across calibration levels, making the calibration procedure difficult to standardize for small-volume fish fecal samples. ExB was therefore designed to improve extraction consistency while allowing extraction efficiency and matrix effects to be evaluated independently. The 24 pooled samples from trial FT2 were processed using ExB. The same procedure described for ExA was followed, except that a 1 mL aliquot of the aqueous supernatant was extracted with 500 µL of MTBE-BPHE. To facilitate phase separation, 600 mg of MgCl_2_ were added, after which the solution was vortexed for 5 min and incubated on ice for 30 min. The organic phase was then collected following centrifugation at 15,000× *g* for 10 min. Three additional serial extractions were subsequently performed using the same volume of MTBE. The organic phases recovered after each extraction step were stored separately in 1.5 mL microcentrifuge tubes and used for method verification by GC-MS. For subsequent SCFA quantification in samples, two serial extractions were performed, and the recovered organic phases were combined and stored until GC-MS analyses.

The main differences between ExB and the initial adaptation ExA have been summarized in Table 1.

### 2.5. GC-MS Analysis

**Method GC1** (TraceGOLD™ TG-5MS column; Thermo Fisher Scientific, Waltham, MA, USA): oven temperature program starting at 36 °C and held for 3 min, increased at 20 °C/min to 44 °C, then at 40 °C/min to 150 °C, and finally at 50 °C/min to 200 °C. Transfer-line and injector temperatures were set at 180 °C and 170 °C, respectively. Helium was used as carrier gas at a flow rate of 1.0 mL/min. Injection volume was 0.5 μL and total run time was 15 min. Detection was performed in full-scan mode (*m*/*z* 35–200, scan time 0.35 s) and SIM mode at *m*/*z* 28, 45, and 60 with a dwell time of 80 ms.

**Method GC2** (TraceGOLD™ TG-5MS column; Thermo Fisher Scientific, Waltham, MA, USA): oven temperature program starting at 32 °C and held for 1 min, followed by an increase at 20 °C/min to 180 °C. Transfer-line and injector temperatures were set at 180 °C and 170 °C, respectively. Helium carrier gas flow was 1.0 mL/min. Injection volume was 0.5 μL and total run time was 10 min. Detection was performed in full-scan mode (*m*/*z* 40–200, scan time 0.2 s) and SIM mode at *m*/*z* 45, 60, and 73 with a dwell time of 400 ms.

**Method GC3** (Nukol™ capillary GC column; Supelco, Bellefonte, PA, USA): oven temperature program starting at 30 °C and held for 2 min, followed by an increase at 40 °C/min to 180 °C. Transfer-line and injector temperatures were maintained at 180 °C and 170 °C, respectively. Helium carrier gas flow rate was 1.0 mL/min. Injection volume was 0.5 μL and total run time was 20 min. Detection was performed in full-scan mode (*m*/*z* 40–160, scan time 0.2 s) and SIM mode at *m*/*z* 45, 60, and 74 with a dwell time of 200 ms.

**Method GC4** (Nukol™ capillary GC column; Supelco, Bellefonte, PA, USA): oven temperature program starting at 35 °C and held for 2 min, followed by an increase at 40 °C/min to 180 °C. Transfer-line and injector temperatures were set at 180 °C and 170 °C, respectively. Helium was used as carrier gas at a flow rate of 1.0 mL/min. Injection volume was 1.0 μL, followed by a 2.0 μL hexane column wash after each injection. Total run time was 15 min. Detection was performed in full-scan mode (*m*/*z* 40–160, scan time 0.2 s) and SIM mode at *m*/*z* 45 and 60 with a dwell time of 400 ms.

Fragment ions were selected based on *m*/*z* ratios minimally affected by matrix background and showing stable and reproducible responses across analyses, thereby enabling linear calibration in standards. For each method, the most abundant fragment ion was selected for quantification to maximize sensitivity. Compound identification was achieved in full-scan mode by matching retention times and mass spectra with those of reference standards. The parameters of each GC-MS have been summarized in Table 2. Following chromatographic optimization, GC4 was selected as the final GC–MS method for quantitative SCFA analysis.

### 2.6. Calibration and Matrix Effect

SCFA recovery from the aqueous phase into MTBE was evaluated by comparing the peak areas obtained for each compound across the four serial extractions. To assess repeatability, two samples were analyzed before and after spiking with standard solutions of each acid. Calibration curves were generated for each compound using pure standards dissolved in MTBE.

To evaluate matrix effects, one sample was lyophilized for four days to promote the removal of endogenous SCFAs. Analysis of the lyophilized blank matrix confirmed the absence of detectable butyric and propionic acids. A small residual acetic acid signal was still observed; however, its concentration was substantially lower than that measured in the biological samples and was therefore considered negligible for the intended analytical application. Water was then added to restore the original sample weight prior to lyophilization, and a solid–liquid extraction was performed following the same procedure used for the other samples to obtain the aqueous phase. Aliquots of the recovered aqueous extract were subsequently spiked with standard solutions at increasing concentrations (acetic acid, 5.44–87.1 μM; propionic acid, 0.275–4.40 μM; butyric acid, 0.675–10.8 μM). One aliquot was kept unspiked and used as a blank control. Each aliquot was then extracted with MTBE according to the corresponding extraction method. Peak areas for each compound were determined by GC–MS and compared with the corresponding peak areas obtained from pure standards, expressed as ratios.

### 2.7. Calculation of Correction Factors for ExB

For extraction method ExB, calibration was performed using pure standard calibration curves rather than matrix-matched standards, as previously done for ExA. Matrix effects were evaluated separately, following the same procedure described for ExA, and used as correction factors for the pure standard calibration curves to compensate for signal overestimation caused by matrix effects.

The extraction efficiency of SCFAs into MTBE was evaluated on three samples subjected to three consecutive extraction steps using ExB. Each extract recovered during the serial extraction procedure was analyzed by GC–MS to determine peak areas. The ratios between extraction steps were then used to estimate recovery rates and calculate the overall recovery obtained after two extraction steps. Unlike ExA, the improved extraction consistency achieved with ExB allowed extraction efficiency to be quantified independently from matrix effects. Consequently, calibration in pure solvent combined with experimentally determined extraction correction factors was considered a more robust and practical approach than matrix-matched calibration for this optimized procedure.

### 2.8. Determination of LOD and LOQ

Limit of detection (LOD) and limit of quantification (LOQ), expressed as µg/mL in the final extract, were calculated according to the ICH Q2(R1) guideline using the calibration curves obtained for each SCFA quantified with ExB. LOD and LOQ were calculated as (3.3σ)/S and (10σ)/S, respectively, where σ represents the standard deviation of the response, estimated from the residual standard deviation of the regression, and S corresponds to the slope of the calibration curve.

## 3. Results and Discussion

### 3.1. Development of the GC-MS Method

#### 3.1.1. GC-MS Method Optimization

**Figure 1:**
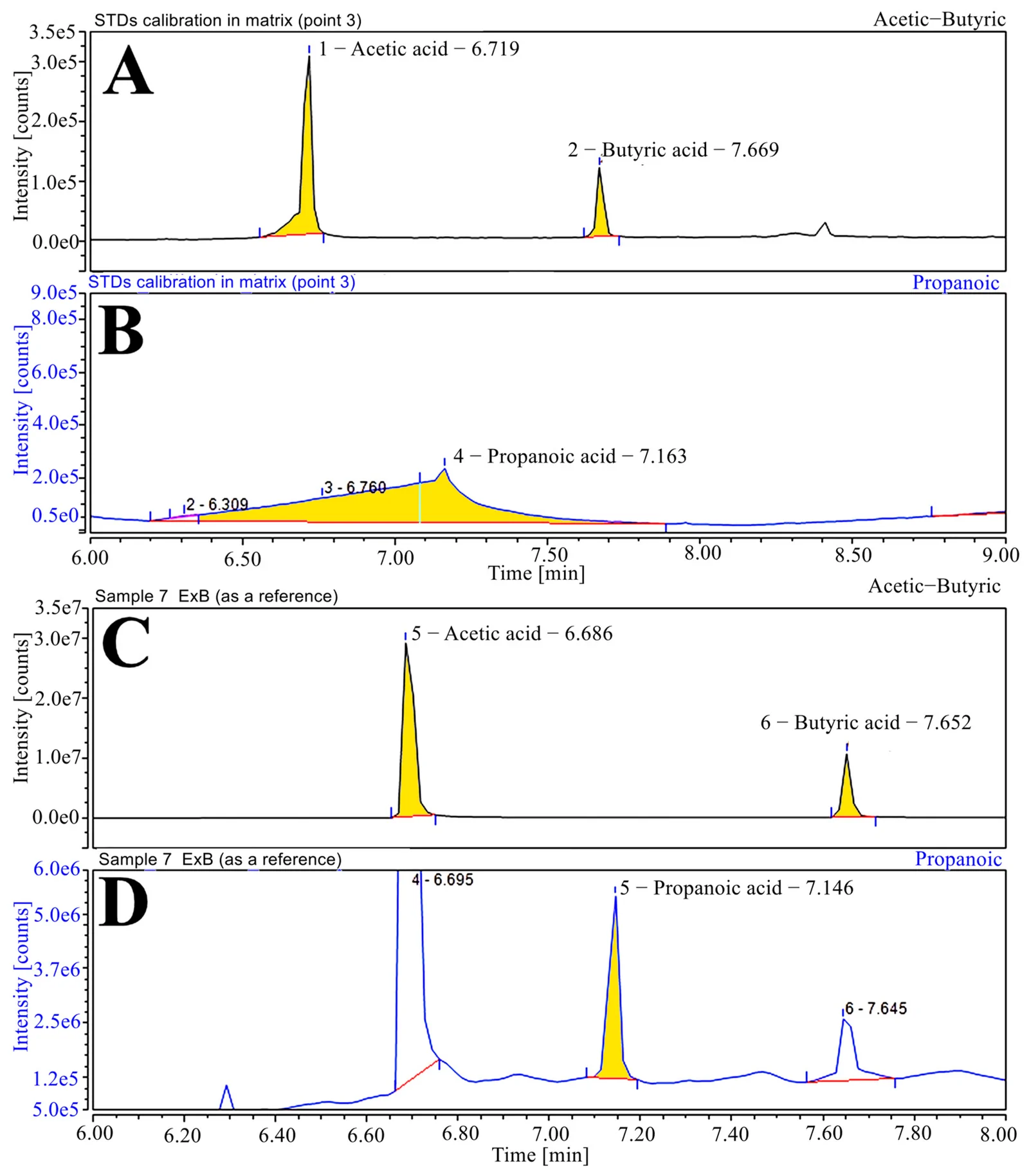
Representative chromatograms obtained from spike-in calibration assays. (**A**,**B**) Chromatograms generated using method ExA after spiking the sample matrix with SCFA standards. (**A**) Ion *m*/*z* 60, used for the quantification of acetic and butyric acids; (**B**) ions *m*/*z* 45 and 73. Peak symmetry indices were 0.93 for acetic acid and 0.95 for butyric acid. In contrast, the propionic acid peak (highlighted) exhibited an irregular shape, preventing reliable quantification. (**C**,**D**) Chromatograms obtained using method ExB with matrix-based calibration. Peak symmetry indices were 1.09 for acetic acid, 1.30 for propionic acid, and 0.96 for butyric acid, indicating improved chromatographic performance compared with ExA (highlighted), (**C**) ion *m*/*z* 60; (**D**) ion *m*/*z* 45.

Calibration solutions containing pure SCFA standards were analyzed by GC–MS using the TG-5MS column. This column did not allow adequate separation of the three acids, as acetic and propionic acids co-eluted with the solvent peak. In contrast, the Nukol column, characterized by a stationary phase composed of acid-modified polyethylene glycol, enabled the separation of all analytes using method GC3 (Figure 1A,B).

The TG-5MS column was initially selected as a general-purpose GC column to evaluate the extraction procedure and assess whether matrix-derived compounds co-extracted with the target analytes could interfere with the analysis. Such compounds were expected to exhibit stronger retention on the more polar Nukol column, potentially affecting chromatographic separation. As no relevant matrix interferences were observed, the Nukol column was subsequently adopted. Its acid-modified polyethylene glycol stationary phase provides stronger interactions with short-chain fatty acids, resulting in improved retention and resolution of these highly polar analytes. Consequently, the co-elution of acetic and propionic acids with the solvent peak was eliminated, making the Nukol column more suitable for the final analytical method.

Further optimization of the chromatographic conditions led to the development of method GC4, which provided improved chromatographic separation, with greater retention time stability and improved peak symmetry for all three analytes (Table 3, Figure 1C,D). Clear separation based on retention times with minimal deviation was observed when pure standard solutions were analyzed (Table 3; Supplementary Tables S1–S12).

Furthermore, analysis of the biological matrix from trial FT1 showed retention times and deviations comparable to those obtained with pure standards (Table 3), suggesting that the sample matrix did not significantly affect SCFA separation.

#### 3.1.2. Additional Methodological Considerations

Optimization of the extraction procedure to resolve issues related to extractant recovery also included several modifications to the overall analytical workflow aimed at improving method reliability. The method was adapted from Yao et al. [16], who used 200 μL of plasma and 24 μL of internal standard (IS) dissolved in 2 N HCl. The acidic conditions promote the protonation of short-chain fatty acids (SCFAs), reducing their polarity and enhancing their partitioning into the organic methyl tert-butyl ether (MTBE) phase. The authors optimized the organic to aqueous phase ratio for plasma samples, enabling the recovery of approximately 60 μL of the MTBE phase for subsequent LC-MS/MS analysis. Similarly, the extraction volumes were adjusted to account for the characteristics of our sample matrix. We found that diluting the sample to the proposed final concentration (1 g in 10 mL) provided the most consistent recovery of the organic phase. Increasing the dilution twofold did not further improve the recovered extract volume or extraction consistency. However, the volume of the recovered organic phase varied among samples.

Therefore, an optimization was realized in the ExB method: phase separation was increased by salt addition to the water phase, allowing more consistent volume or recovery. Further, calibration of the second set of processed samples (FT2, ExB) was performed with pure standards rather than matrix-matched standards, as previously applied for FT1 samples processed with ExA. This modification enabled better discrimination among variability sources related to extraction yield, calibration linearity, and matrix effects.

When applying this workflow to quantify SCFAs in samples originating from the same feeding trial, or from multiple feeding trials within the same experiment, we recommend consistently applying a single calibration strategy to all samples. Similarly, the injection volume was increased from 0.5 µL for FT1 samples to 1.0 µL for FT2 samples to improve sensitivity for propionic acid, which was present at low abundance in our samples. This modification improved propionic acid detection while maintaining satisfactory chromatographic quality.

To provide more robust validation of method suitability under different sampling conditions, we further recommend evaluating matrix effects and extraction recovery using a representative number of samples according to the experimental design. In terms of analytical throughput, the method involves relatively short GC–MS runs of approximately 15 min per sample. Considering at least three replicate analyses per sample to estimate mean values and variability, the workflow allows the processing of approximately 12 samples per day using a single injector. This estimate also accounts for the inclusion of two blank runs after each sample and daily verification of the calibration curve as a quality control measure.

### 3.2. Extraction of SCFAs and Matrix Effect Evaluation

#### 3.2.1. Extraction Method ExA

The initially established extraction method (ExA) allowed the recovery of only 24% to 60% of the MTBE extractant volume when biological samples were used as the starting material. In contrast, application of the same method to aqueous solutions containing pure SCFA standards resulted in the recovery of approximately 80% of the added MTBE on average (Table 4, Supplementary Table S13).

These findings indicate a matrix-associated effect on phase separation and extractant recovery, resulting in substantial variability in MTBE recovery among biological samples. The underlying physicochemical mechanism was not directly investigated. Importantly, the addition of MgCl_2_ markedly improved the extraction procedure, increasing MTBE recovery from 40.1% to 85.3% and reducing the CV from 29.8% to 5.5%, consistent with improved phase separation through a salting-out effect. Although the specific matrix components responsible were not investigated, the effect is consistent with the presence of endogenous constituents that alter phase behavior. This issue was effectively compensated by the addition of salt, which enhanced phase separation through a salting-out effect and improved extraction reproducibility. MTBE exhibits a water solubility of approximately 26 g/L at 20 °C (about 4.3% *w*/*w*), while water is soluble in the organic phase at around 1.4%. This mutual solubility is higher than that observed for many other ethers and generally promotes efficient mixing, facilitating the extraction of low- to moderately polar compounds into the organic phase. However, the incorporation of water into the organic phase may also contribute to inconsistent phase separation in complex biological matrices.

In addition, matrix components may act as surfactants, further hindering the separation of the aqueous and organic phases after centrifugation. Biphenyl, which is exclusively soluble in the organic phase, was therefore used as a surrogate standard to monitor extraction efficiency and recovery, even under conditions in which the biological matrix could influence phase partitioning. Nevertheless, despite the presence of the standard, the variability in extractant recovery may still have introduced bias in the quantification of the target compounds. This issue is particularly critical for SCFAs, which are intrinsically difficult to isolate and quantify due to their volatile nature and their rapid uptake by host cells within the gut environment [5].

When evaluating matrix effects for ExA, an overestimation of SCFAs concentrations ranging from 14.7% to 37.6% was observed, depending on the analyte quantified (Table 5).

**Figure 2:**
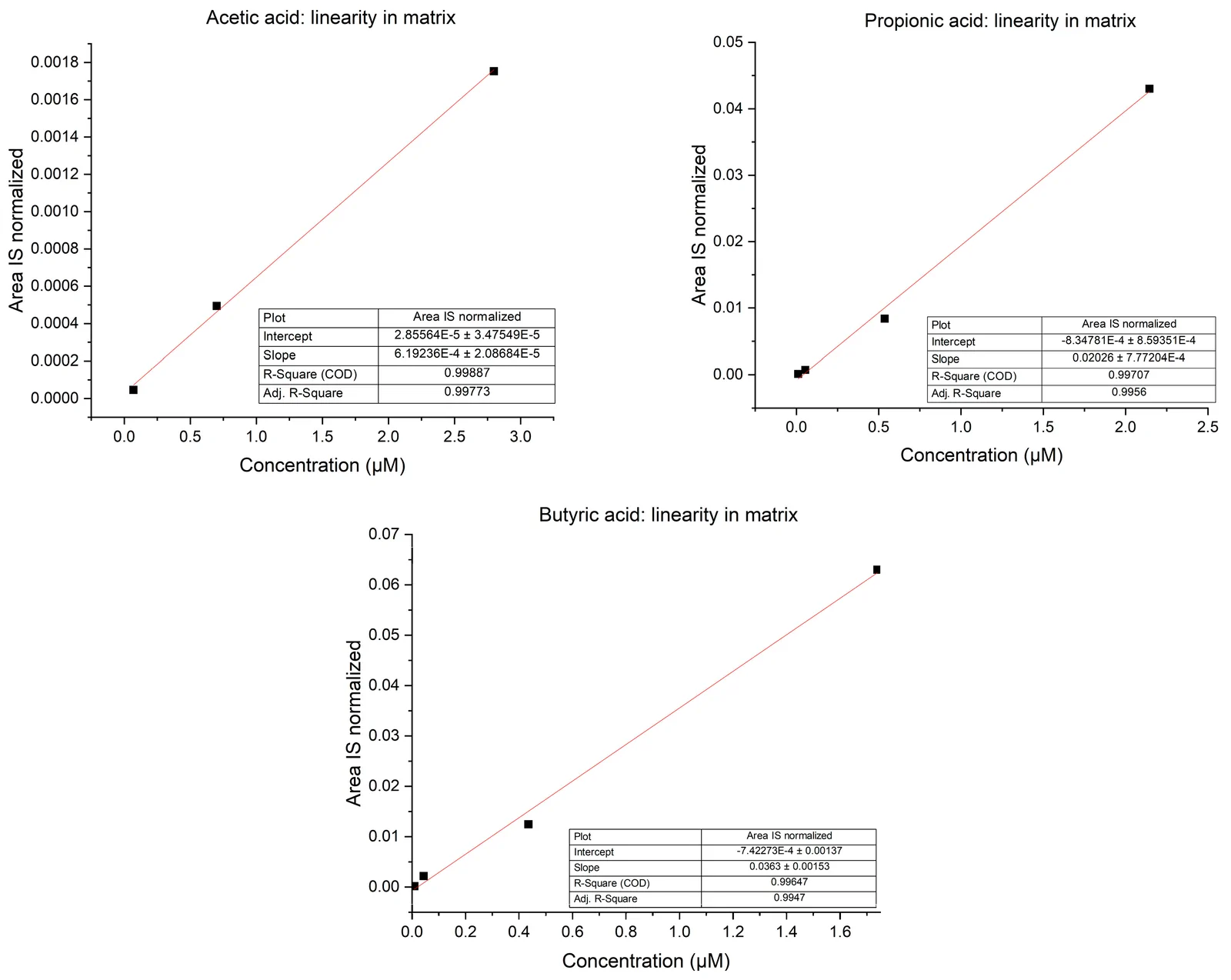
Calibration plots for SCFAs in matrix using method ExA. Linear calibration curves of internal standard–corrected peak areas obtained for quantified SCFAs in matrix extracts processed with ExA and spiked with acid standards are shown. **Note:** Error bars are not shown because replicate measurements were not performed for each calibration point at this stage of method development. At this preliminary stage, satisfactory linearity was considered the primary criterion for evaluating the analytical response and suitability of the method; consequently, variability estimates are not available for the individual points shown.

**Figure 3:**
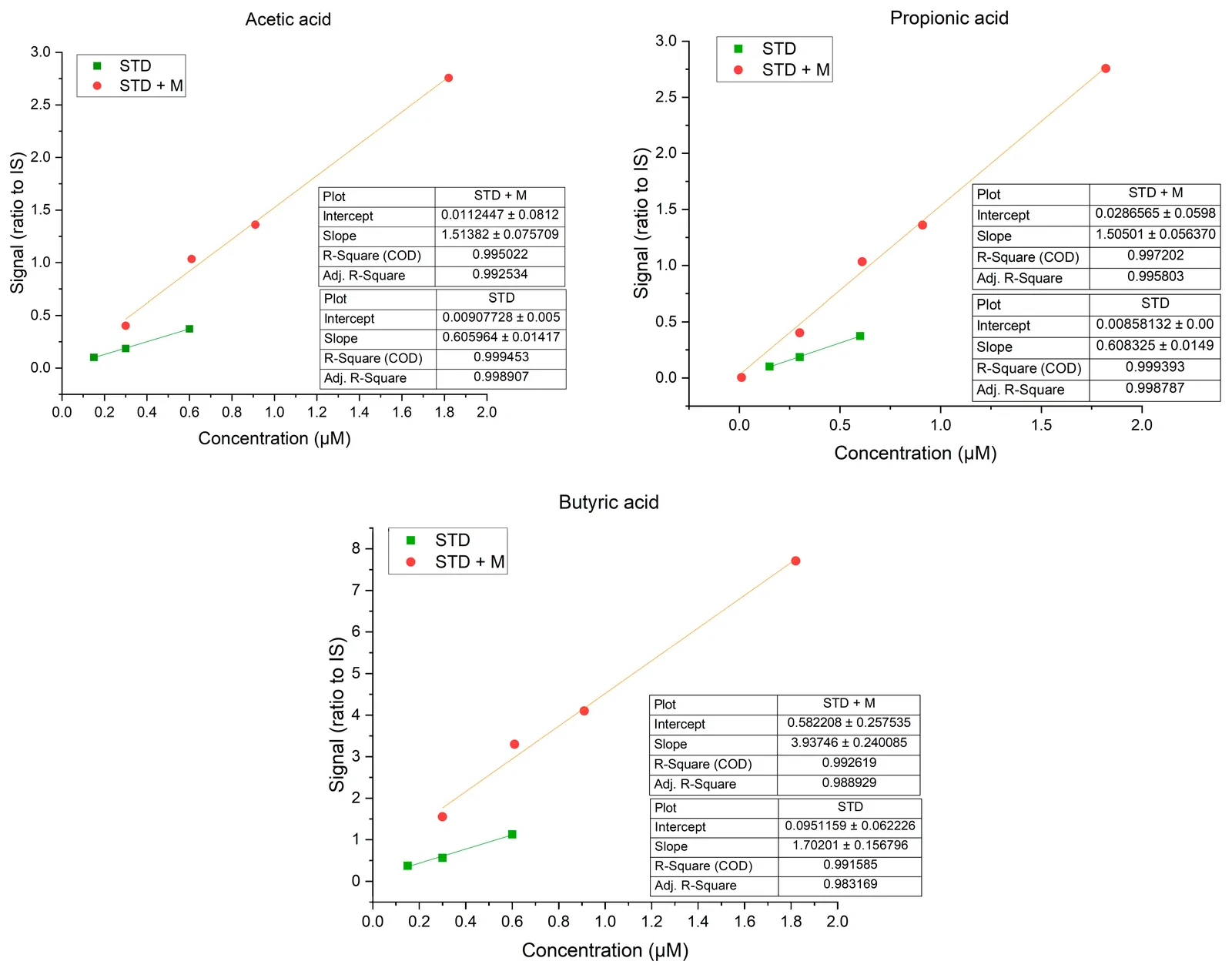
Assessment of matrix effects for method ExA. Plots show the relationship between SCFA concentration (µg/mL) and internal standard–normalized peak areas, used to evaluate matrix effects for each quantified compound. **Note:** Error bars are not shown because replicate measurements were not performed for each calibration point at this stage of method development. At this preliminary stage, satisfactory linearity was considered the primary criterion for evaluating the analytical response and suitability of the method; consequently, variability estimates are not available for the individual points shown.

Furthermore, when ExA was used for sample extraction, samples diluted 20-fold in fresh MTBE prior to GC-MS analysis showed satisfactory calibration linearity for all three compounds. Calibration was performed using matrix-matched standards based on the peak areas obtained for each analyte (Figure 2), while injection bias was corrected through internal standard spiking prior to injection. The resulting linearity coefficients (R^2^) demonstrated a strong linear correlation between peak area and SCFA concentration, enabling reliable quantification of the three acids in the samples while accounting for the observed matrix effects (Figure 3).

Although this extraction method enabled linear quantification of the target compounds (R^2^ ≥ 0.9965) (Figure 2), its main limitation was the poor recovery of the organic solvent after extraction, combined with substantial sample-to-sample variability in recovered volumes. This issue could reduce analytical throughput and decrease confidence in quantification, particularly when processing large numbers of sensitive biological samples.

To overcome these limitations, an improved extraction method (ExB) was developed and applied to samples collected at the end of trial FT2.

#### 3.2.2. Adapted Extraction Method ExB

**Figure 4:**
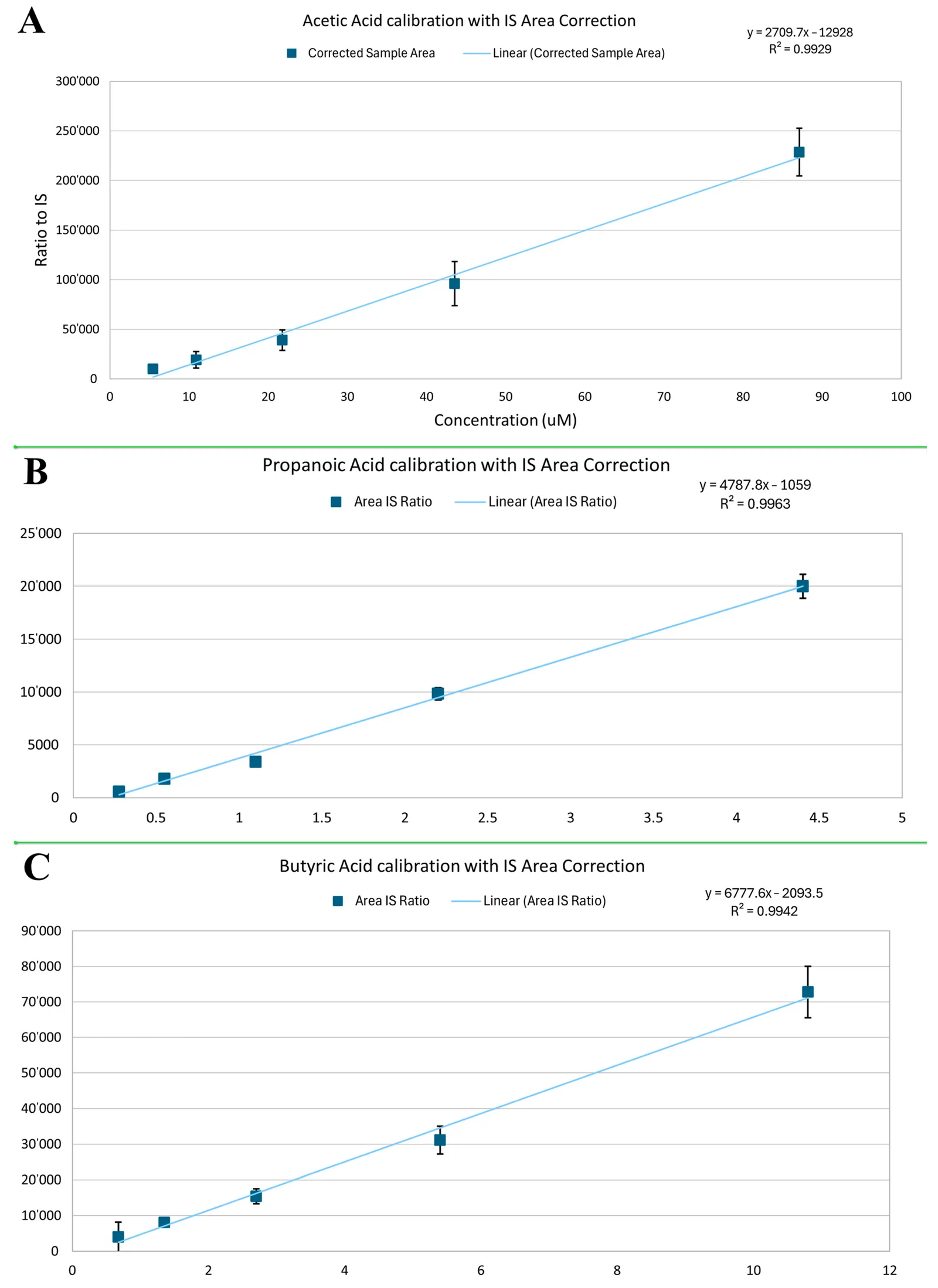
Calibration curves of acetic acid (**A**), propionic acid (**B**), and butyric acid (**C**) generated using internal standard (IS) area correction for the quantification of ExB samples.

When ExB was applied to samples from trial FT2, the organic extraction phase could be recovered almost completely when extracting either aqueous pure standard solutions, or the sample matrix, with similar recovered volumes across processed samples. This high recovery is likely due to the higher MTBE: water ratio in the liquid–liquid extraction phase, and the addition of MgCl_2_, which promotes phase separation and improves organic phase retrieval. It appears that these modifications in the extraction method were able to successfully counteract the surfactant effect of the matrix on the MTBE-water partitioning. Pure standard solutions and spiked sample matrices were compared to evaluate retention time consistency (Table 3), revealing differences of no more than 1.5 s. Extraction efficiency, matrix effects, and linearity coefficients are reported in Table 6 and Supplementary Tables S5–S12. Matrix effects were calculated as a percent ratio between the signal (area) of pure standard concentration tested and that same concentration spiked in the depleted sample after lyophilization. Extraction efficiency (recovery) increased with the carbon chain length of the organic acids. It is important to clarify that the results of this method in terms of detected concentrations cannot be used to compare the methods because they are representative of different feeding trials and subject to biological variability. ExB method can be compared to ExA as a functional optimization to enhance the recovery consistency of MTBE and to have further control over matrix effect from one sample set to the others. Figure 4 shows the calibration curves for acetic, propionic, and butyric acids obtained using internal standard (IS) area correction.

#### 3.2.3. ExB Method Considerations and Limitations

Two serial extractions, using half the MTBE volume applied in ExA, recovered most of the acetic acid, although detectable amounts were still present in a third extraction (Table 6). The comparatively lower and more variable recovery of acetic acid is consistent with its higher water solubility and greater tendency to remain in the aqueous phase compared with the longer-chain SCFAs [17]. Consequently, its recovery after two liquid–liquid extraction steps is more sensitive to small variations in phase separation and extraction conditions. While this did not constitute an issue with quantification in our case, due to the high abundance of acetic acid in our samples, it might present a limitation in the case of samples with low acetic acid concentrations near the LOD. In such cases, we recommend first optimizing the extraction procedure for the specific sample matrix. If sufficient sample material is available, increasing the sample weight while maintaining an appropriate sample-to-solvent ratio is preferable to applying a universal correction factor. Where optimization is not sufficient, an additional serial extraction may be considered to maximize acetic acid recovery. In particular, increasing the sample weight while maintaining an appropriate sample-to-solvent ratio would be preferable, as acetic acid is highly soluble in water. This approach is more appropriate than applying a universal correction factor, whose accuracy may vary depending on the analyte concentration and sample matrix.

Propionic and butyric acids were almost entirely extracted in the first two steps, with butyric acid not even detected in the third serial extraction. The method showed a maximum matrix effect of 17.4% for butyric acid and an R^2^ consistently greater than 0.992. Extraction efficiency, representing the partitioning of SCFAs between water and MTBE, was used to derive correction factors for SCFA quantification in samples.

To the best of our knowledge, studies evaluating the extraction and quantification of SCFAs from fish fecal samples are currently lacking. Consequently, meaningful comparisons are limited, highlighting the need for methodological developments such as the one presented in this work. Isotope-labeled internal standards could further improve quantitative accuracy by more effectively compensating for matrix effects, extraction losses, and instrumental variability. Their use would be particularly valuable when applying the method to different biological matrices or experimental conditions, where matrix composition may vary. However, in the present study, the observed matrix effects were low (<17.4%) and the analytical performance of the method, including recovery, linearity, and precision, was satisfactory using the selected internal standard. Therefore, although isotope-labeled internal standards may further enhance method robustness, their inclusion was beyond the scope of this study.

Overall, ExB exhibited reduced matrix effects and improved linearity compared with ExA. This enabled the quantification of butyric acid over a lower concentration range, thereby increasing sensitivity toward this compound (Table 7). LOD and LOQ values, expressed as micromolar concentrations in the injected samples, were obtained for each calibration curve and are reported in Supplementary Materials Tables S5–S12. In addition, the combination of ExB with GC4 improved analyte peak symmetry and retention time stability.

Considering the minimum and maximum concentrations of the three SCFAs measured in FT1 and FT2, some observations suggest that the differences between the two trials are unlikely to be explained solely by extraction efficiency. In particular, FT2 samples, which were processed using ExB, showed markedly lower butyric acid concentrations (Table 7), although butyric acid exhibited the highest extraction recovery among the analytes investigated (95.5%; Table 6). Conversely, higher concentrations of both propionic and acetic acids were detected in FT2 using the same extraction method (Table 7), despite their lower extraction recoveries compared with butyric acid (80.9% and 44.9%, respectively; Table 6). This pattern is therefore consistent with a contribution of biological variability rather than with a systematic effect of extraction efficiency alone.

Nevertheless, FT1 and FT2 were independent feeding trials conducted on fish of different sizes, although the same dietary treatments were used, and the corresponding samples were processed using different extraction and calibration workflows. Specifically, FT1 samples were analyzed using ExA with matrix-matched calibration, whereas FT2 samples were analyzed using ExB with pure-solvent calibration combined with experimentally determined correction factors. Therefore, the differences in SCFA concentration ranges observed between FT1 and FT2 cannot be attributed unequivocally either to biological variation or to the analytical procedure. The values reported in Table 7 are intended primarily to illustrate the concentration ranges encountered during method development and should not be interpreted as a direct quantitative comparison between FT1 and FT2. A paired analysis of the same biological samples using both analytical workflows would be required to formally distinguish methodological from biological sources of variation. Since this study was designed to optimize the extraction procedure, only a limited number of samples was included, and the sample size was not intended for biological comparisons. Future studies involving larger sample sets will allow a more comprehensive evaluation of the observed variability and help determine whether it reflects genuine biological differences or the presence of outliers.

#### 3.2.4. Considerations About Sample Pooling

In our experience, the W/V ratios of our extraction method allow for extraction from 0.8 to 1 g of starting material to stay within manageable working volumes. Here, 2–3 individual samples were pooled when necessary to reach an adequate weight of starting material to extract from (1 g). Pooling may be considered a limitation in quantitative analyses due to it potentially reducing inter-individual variability and masking extremes. Aguirre et al. [18] compared SCFA and branched-chain fatty acid quantification in individual and pooled fecal samples and found that pooling reduced the impact of inter-individual variability. The authors suggested that, in studies primarily aimed at evaluating dietary effects at the group level, pooling samples may help minimize the influence of atypical responses from individual animals. Therefore, sample pooling represents an important methodological trade-off: while it reduces biological variability and may improve the detection of group-level effects, it also masks individual variability, which may be relevant depending on the objectives of the study.

Moreover, the necessity to pool, however, depends on the amount of material that is sampled by each individual, which in turn depends on the size and feeding status of the fish at the time of sampling. We recommend that at least 1 g of material is sampled where possible, and if not, pooling be performed consistently and keeping in mind the experimental design, the number of factors being tested and the research question.

#### 3.2.5. Estimation of Extraction Method Throughput

Extraction method throughput was not formally validated. However, based on our laboratory experience, we estimate that 6–10 samples can be processed in parallel by a single operator and the full extraction protocol can be completed within 3–4 h, of which 1.5–2 h are inactive time of incubations on ice, centrifugation and vortexing. These estimations are based on the use of a horizontal vortex that allows the vortexing of two falcons at a time, a 16-tube centrifuge rotor for falcons and a 24-tube microcentrifuge. The time and number of samples processed may vary depending on the infrastructure of the executing laboratory. While more than 10 units can be processed simultaneously, we believe that increasing the throughput beyond 10 may introduce a risk of SCFA evaporation due to increased processing time at the initial steps of the solid–liquid extraction. Ultimately, the decision comes down to the total number of samples to be processed, as well as time and infrastructure availability.

## 4. Conclusions

We analyzed two sets of samples obtained from independent feeding trials that used the same dietary treatments and were conducted under comparable husbandry conditions. Following extraction of the first sample set using an adaptation of a previously published method, we identified several limitations that hindered efficient and time-efficient SCFA quantification in this type of biological sample. At present, only a limited number of published methods are suitable for the analysis of small fish fecal samples, in which SCFA concentrations are expected to be low and the amount of available material is highly restricted.

The newly developed extraction method, ExB, produced more consistent and repeatable results. In addition, ExB treats matrix effects and extraction yield as independent parameters, enabling a more rigorous assessment of extraction performance. Although matrix effects observed with both extraction methods did not compromise calibration linearity, ExB substantially reduced matrix effects while improving extraction efficiency.

At the chromatographic level, the use of a Nukol column enabled effective separation of three SCFAs under investigation without the need for derivatization. In this study, we focused on the extraction and quantification of acetic, propionic, and butyric acids because these SCFAs are associated with specific microbial communities within the gut, and are the compounds of interest within the scope of our present line of research. While this may limit the current applicability of the method, future studies should evaluate whether the optimized ExB protocol can be adapted for the quantification of additional SCFAs, including valeric acid, isovaleric acid, and isobutyric acid. Such applications will require further optimization and validation.

The method also warrants further investigations. In particular, butyric acid showed higher recovery than propionic and acetic acids in MTBE. Future studies may further evaluate the applicability of this method for the quantification of additional volatile fatty acids and for use with other comparable biological or food-related matrices. In addition, although the present validation demonstrated satisfactory analytical performance, including low matrix effects, good linearity, and adequate recovery, further validation should be undertaken to assess long-term reproducibility, precision, accuracy, and robustness. Such studies will further support the applicability of the method for routine analysis and its implementation across different sample types and experimental settings.

## Figures and Tables

**Table 1:** Main steps and rationale of extraction methods ExA and ExB.

Parameter	ExA—First Method Adaptation	ExB—Second Method Adaptation
Based on	Adapted from Yao et al. [16]	Modified version of ExA
Reason for development	Selected due to similar physical characteristics of the samples and suitability for SCFA quantification	Developed to address practical issues encountered with ExA during FT1
Samples processed	18 pooled samples (Trial FT1)	24 pooled samples (Trial FT2)
Starting material	1 g partially thawed fecal matter	1 g partially thawed fecal matter
Extraction solvent	10 mL of 1.2 M HCl	Same as ExA
Initial extraction	Vortex 10 min	Same as ExA
First centrifugation	3500× *g*, 4 °C, 10 min	Same as ExA
Volume of aqueous phase used	Entire recovered aqueous phase	1 mL aliquot of aqueous supernatant
Organic extraction solvent	1 mL MTBE-BPHE	500 µL MTBE-BPHE
Vortex after organic solvent addition	2 min	5 min
Salt addition	None	600 mg MgCl_2_
Ice incubation	None	30 min
Second centrifugation	3500× *g*, 4 °C, 5 min	15,000× *g*, 10 min
Serial extractions	Three additional extractions with 200 µL fresh MTBE	Three additional extractions with 500 µL MTBE (same volume as first extraction) for method verification
Storage of extracts	Organic phases from each extraction stored separately	Organic phases stored separately in 1.5 mL tubes for method verification
Routine quantification approach	Individual extracts analysed separately	Two serial extractions performed; organic phases combined before storage and GC-MS analysis
Main objective	Initial adaptation for SCFA extraction	Improved phase separation, extraction efficiency, and simplified workflow

**Table 2:** Summary of four GC-MS methods for the separation and detection of SCFAs.

Parameter	GC1 (TraceGOLD)	GC2 (TraceGOLD)	GC3 (Nukol)	GC4 (Nukol)
Column	TraceGOLD	TraceGOLD	Nukol	Nukol
Initial oven temperature	36 °C	32 °C	30 °C	35 °C
Initial hold	3 min	1 min	2 min	2 min
Ramp program	20 °C/min to 44 °C 40 °C/min to 150 °C 50 °C/min to 200 °C	20 °C/min to 180 °C	40 °C/min to 180 °C	40 °C/min to 180 °C
Final temperature	200 °C	180 °C	180 °C	180 °C
Transfer-line temperature	180 °C	180 °C	180 °C	180 °C
Injector temperature	170 °C	170 °C	170 °C	170 °C
Carrier gas	Helium	Helium	Helium	Helium
Carrier gas flow	1.0 mL/min	1.0 mL/min	1.0 mL/min	1.0 mL/min
Injection volume	0.5 μL	0.5 μL	0.5 μL	1.0 μL
Column wash	None	None	None	2.0 μL hexane after each injection
Total run time	15 min	10 min	20 min	15 min
Full-scan range	*m*/*z* 35–200	*m*/*z* 40–200	*m*/*z* 40–160	*m*/*z* 40–160
Full-scan time	0.35 s	0.2 s	0.2 s	0.2 s
SIM ions	28, 45, 60	45, 60, 73	45, 60, 74	45, 60
SIM dwell time	80 ms	400 ms	200 ms	400 ms

**Table 3:** Retention times (min) of SCFAs analyzed on a Nukol column using the GC-MS method GC4. Retention times obtained in biological matrix samples are reported for both extraction methods as mean ± SD of 3 replicates.

Compound	Standards in MTBE	Standards in Matrix (ExA)	Asymmetry (ExA)	Standards in Matrix (ExB)	Asymmetry (ExB)
**ACETIC ACID**	6.712 ± 0.009	6.722 ± 0.014	0.89 ± 0.0487	6.714 ± 0.009	1.02 ± 0.0583
**PROPIONIC ACID**	7.166 ± 0.007	7.166 ± 0.014	0.56 ± 0.2880	7.160 ± 0.007	0.865 ± 0.0975
**BUTYRIC ACID**	7.672 ± 0.008	7.692 ± 0.008	0.74 ± 0.0932	7.667 ± 0.003	1.03 ± 0.0618

**Table 4:** MTBE recovery following liquid–liquid extraction in 3 replicates using methods ExA and ExB, expressed as recovered volume and percentage of the initial added volume (mean ± SD), together with the coefficient of variation (CV). ExA 1–3 indicate the three serial extraction steps, while “Tot.” represents the total MTBE volume recovered across all serial extractions combined.

	Added MTBE (µL)	Recovered MTBE (µL)	Recovered MTBE (%)	CV (%)
**ExA 1°**	1000	293 ± 200	29.3 ± 20.0	68.3
**ExA 2°**	200	102 ± 87	51.2 ± 43.6	85.0
**ExA 3°**	200	165 ± 113	82.6 ± 56.5	68.4
**ExA tot.**	1400	561 ± 167	40.1 ± 11.9	29.8
**ExB tot.**	1000	853 ± 47	85.3 ± 4.7	5.5

Recovered MTBE (%) = [Recovered MTBE (µL)/Added MTBE (µL)] × 100.

**Table 5:** Matrix effect (mean ± SD, *n* = 3) calculated for each SCFA following extraction using method ExA.

Compound	Matrix Effect
Acetic acid	14.7 ± 1.5%
Propionic acid	18.4 ± 2.4%
Butyric acid	37.6 ± 4.1%

**Table 6:** Cumulative recovery (%) of SCFAs in the MTBE organic phase obtained using ExB from two consecutive extraction steps (*n* = 3), together with the percentage of signal overestimation caused by matrix effects and the corresponding calibration linearity coefficients (R^2^).

Compound	Serial Extraction Percent in MTBE (%)	Matrix Effect (%)	Linear Model 5 Points Fit (R^2^)
Acetic acid	44.9 ± 7.8	11.8 ± 0.292	0.9928
Propionic acid	80.9 ± 2.88	4.80 ± 0.567	0.9963
Butyric acid	95.5 ± 9.24	17.4 ± 5.88	0.9942

**Table 7:** Minimum and maximum SCFA concentrations (µmol/g fecal matter) extracted and quantified using methods ExA and ExB. The reported calibration ranges correspond to the peak areas obtained from samples processed with each extraction method.

Compound	ExA	ExB
Butyric acid	0.215–2.362	0.007–0.103
Propionic acid	0.100–1.318	0.497–9.539
Acetic acid	2.431–11.386	9.539–38.786

## Data Availability

The original contributions presented in this study are included in the article/Supplementary Materials. Further inquiries can be directed to the corresponding authors.

## Supplementary Materials

The following supporting information can be downloaded at: https://www.mdpi.com/article/10.3390/metabo16090636/s1, Table S1: calibration in matrix for acetic acid, method ExA; Table S2: calibration in matrix for propionic acid, method ExA; Table S3: calibration in matrix for butyric acid, method ExA; Table S4: biphenyl injection bias correction in matrix, method ExA; Table S5: matrix effect estimation for acetic acid using method ExB; Table S6: matrix effect estimation for propionic acid using method ExB; Table S7: matrix effect estimation for butyric acid using method ExB; Table S8: ethyl butyrate reference spike-in injections in matrix with method ExB; Table S9: pure acetic acid standard calibration and correction with ethyl butyrate as internal standard (IS) for method ExB; Table S10: pure propionic acid standard calibration and correction with ethyl butyrate as internal standard (IS)for method ExB; Table S11: pure butyric acid standard calibration and correction with ethyl butyrate as internal standard (IS) for method ExB; Table S12: ethylbutyrate alone used as internal standard (IS) for injection bias correction in method ExB; Table S13: Volumes of recovered organic phase and percentage of recovery per each sample, extracted with either the ExA or ExB method.

## References

[1] Nagatomo R., Ichikawa A., Kaneko H., Inoue K. (2024). Comparison of 3-nitrophenylhydrazine, O-benzyl hydroxylamine, and 2-picolylamine derivatizations for analysis of short-chain fatty acids through liquid chromatography coupled with tandem mass spectrometry. Anal. Sci..

[2] Czarnowski P., Mikula M., Ostrowski J., Żeber-Lubecka N. (2024). Gas chromatography–mass spectrometry-based analyses of fecal short-chain fatty acids (SCFAs): A summary review and own experience. Biomedicines.

[3] Rimoldi S., Di Rosa A.M., Oteri M., Chiofalo B., Hasan I., Saroglia M., Terova G. (2024). The impact of diets containing *Hermetia illucens* meal on the growth, intestinal health, and microbiota of gilthead seabream (*Sparus aurata*). Fish. Physiol. Biochem..

[4] Wang M., Wichienchot S., He X., Fu X., Huang Q., Zhang B. (2019). In vitro colonic fermentation of dietary fibers: Fermentation rate, short-chain fatty acid production and changes in microbiota. Trends Food Sci. Technol..

[5] Sakata T. (2018). Pitfalls in short-chain fatty acid research: A methodological review. Anim. Sci. J..

[6] van Eijk H.M.H., Bloemen J.G., Dejong C.H.C. (2009). Application of liquid chromatography–mass spectrometry to measure short-chain fatty acids in blood. J. Chromatogr. B.

[7] Takasu S., Nagasaki Y., Koizumi J., Koshizuka T., Esaka Y. (2025). Simple and sensitive LC–MS analysis of short-chain fatty acids in cecum content and feces in mice: A derivatization-free approach. J. Chromatogr. A.

[8] Saha S., Day-Walsh P., Shehata E., Kroon P.A. (2021). Development and validation of a LC–MS/MS technique for the analysis of short-chain fatty acids in tissues and biological fluids without derivatisation using isotope labelled internal standards. Molecules.

[9] McKay M.J., Castaneda M., Catania S., Charles K.A., Shanahan E., Clarke S.J., Engel A., Varelis P., Molloy M.P. (2023). Quantification of short-chain fatty acids in human stool samples by LC-MS/MS following derivatization with aniline analogues. J. Chromatogr. B.

[10] de Sena Aquino A.C.M., Azevedo M.S., Ribeiro D.H.B., Costa A.C.O., Amante E.R. (2015). Validation of HPLC and CE methods for determination of organic acids in sour cassava starch wastewater. Food Chem..

[11] Olonimoyo E.A., Amradi N.K., Lansing S., Asa-Awuku A.A., Duncan C.M. (2025). An improved underivatized, cost-effective, validated method for six short-chain fatty organic acids by high-performance liquid chromatography. J. Chromatogr. Open.

[12] De Baere S., Eeckhaut V., Steppe M., De Maesschalck C., De Backer P., Van Immerseel F., Croubels S. (2013). Development of an HPLC–UV method for the quantitative determination of four short-chain fatty acids and lactic acid produced by intestinal bacteria during in vitro fermentation. J. Pharm. Biomed. Anal..

[13] Kim K.-S., Lee Y., Chae W., Cho J.-Y. (2022). An improved method to quantify short-chain fatty acids in biological samples using gas chromatography–mass spectrometry. Metabolites.

[14] Han Y., Du J., Li J., Li M. (2019). Quantification of the organic acids in hawthorn wine: A comparison of two HPLC methods. Molecules.

[15] Zheng X., Chen T., Li W., Wang K., Xue X., Naumovski N., Peng W. (2024). Rapid purification and quantification of intestinal and fecal short-chain fatty acids by solid-phase extraction using bond elut plexa. Separations.

[16] Yao L., Davidson E.A., Shaikh M.W., Forsyth C.B., Prenni J.E., Broeckling C.D. (2022). Quantitative analysis of short-chain fatty acids in human plasma and serum by GC–MS. Anal. Bioanal. Chem..

[17] Ruderman G., Caffarena E.R., Mogilner I.G., Tolosa E.J. (1998). Hydrogen bonding of carboxylic acids in aqueous solutions—UV spectroscopy, viscosity, and molecular simulation of acetic acid. J. Solut. Chem..

[18] Aguirre M., Ramiro-Garcia J., Koenen M.E., Venema K. (2014). To pool or not to pool? Impact of the use of individual and pooled fecal samples for in vitro fermentation studies. J. Microbiol. Meth..

