# Supplementary material for: A Derivatization-Free GC–MS Method for Reliable Quantification of Short-Chain Fatty Acids in Sparus aurata
Source: Metabolites. 2026 Aug 31;16(9):636. doi: 10.3390/metabo16090636 (PMC13609257; doi:10.3390/metabo16090636)

Table S1: calibration in matrix for acetic acid, method ExA

No	Injection Name		Ret.Time min siim60	Area counts*min siim60	Height counts siim60	Amount n.a. siim60	Rel.Area % siim60	Peak Type siim60	Area normalized	Height normalized
7,5	Pure_STD_7.5	0,15	6,72	2825247	112546646,9	n.a.	21,22	BMB	0,10242615	0,1648501
15	Pure_STD_15	0,3	6,721	4900933	212851693,5	n.a.	99,19	BMB	0,18718493	0,3205861
30	Pure_STD_30	0,6	6,723	9808255	212851693,5	n.a.	92,08	BMB	0,37388268	0,3216135
5	23-10_mix_5uL	0,3	6,736	977121,8	428210138,7	n.a.	20,46	BMB*	0,40274244	7,4489659
10	23-10_mix_10uL	0,61	6,72	2855081	139710447,3	n.a.	23,76	BMB	1,03461093	1,9374855
15	23-10_mix_15uL	0,91	6,72	3362119	147451517,7	n.a.	24,81	BMB	1,36059162	2,3945908
30	23-10_mix_30uL	1,82	6,72	4918796	222593662,4	n.a.	26,25	BMB	2,7573541	4,8715113
0	23-10_mix_0uL	*0,01087*	6,72	16551,88	600706,486	n.a.	5,26	MB	0,00514834	0,0073192

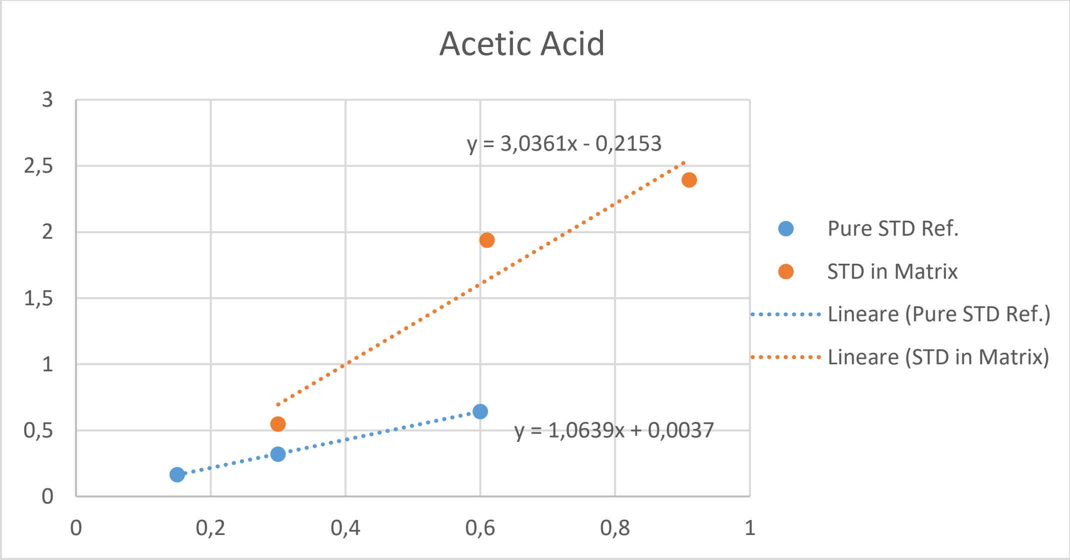

Supplement: Supplementary file 1 [file metabolites-16-00636-s001.zip › Table S1.pdf]
