# Supplementary material for: A Derivatization-Free GC–MS Method for Reliable Quantification of Short-Chain Fatty Acids in Sparus aurata
Source: Metabolites. 2026 Aug 31;16(9):636. doi: 10.3390/metabo16090636 (PMC13609257; doi:10.3390/metabo16090636)

Table S10: pure propionic acid standard calibration and correction with ethyl butyrate as internal standard (IS)for method ExB

Inj. No	Injection Name (Selected Peak)	Type Concentr uM	Ret.Time min Propanoic Propanoic acid	Amount µM Propanoic Propanoic acid	Rel.Area % Propanoic Propanoic acid	Area counts*min Propanoic **Area blanked**	IS RATIO **Area IS Ratio**	RAW Area	BLANK REF AREA 730 **Corrected Sample Area**
105	STDs point 1	**4,4**	7,163	n.a.	10,08	**19997,634**	**0,16510539**	20727,6341	**19997,6341**
107	STDs point 2	**2,2**	7,163	n.a.	9,89	**8360,0766**	**0,081111195**	9090,0766	**9824,221853**
109	STDs point 3	**1,1**	7,163	n.a.	4,91	**2727,3370**	**0,027817998**	3457,337	**3369,327566**
111	STDs point 4	**0,55**	7,147	n.a.	2,14	**1420,0997**	**0,014690539**	2150,0997	**1779,324167**
113	STDs point 5	**0,275**	7,163	n.a.	1,12	**435,25560**	**0,0045407289**	1165,2556	**549,9749784**

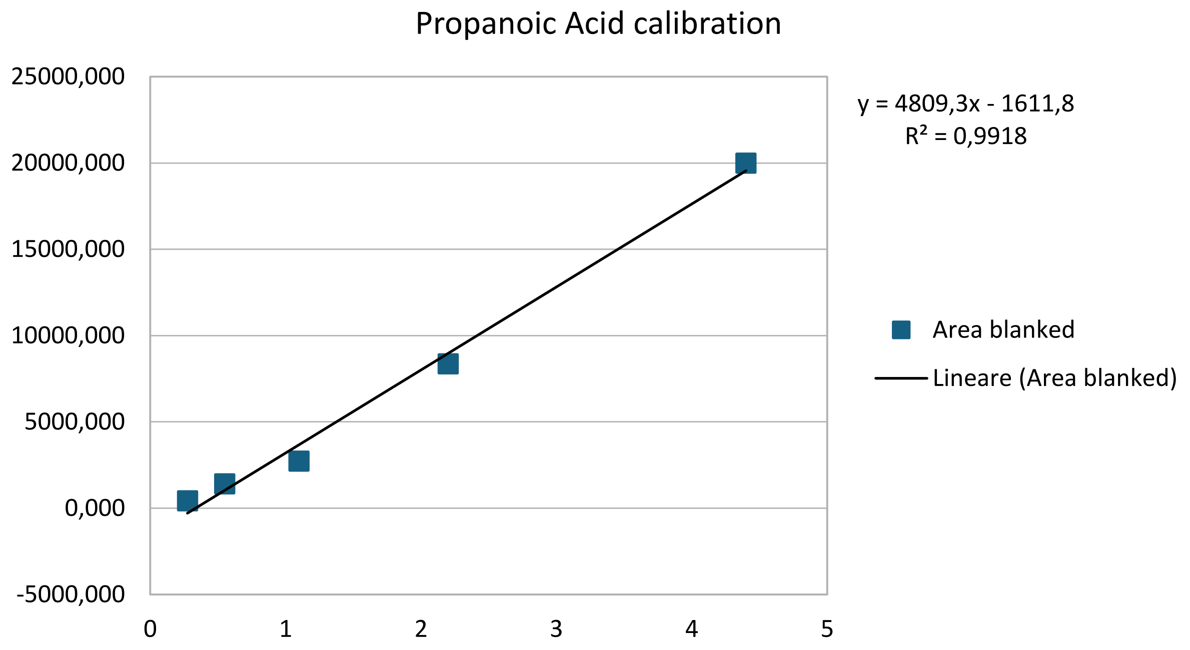

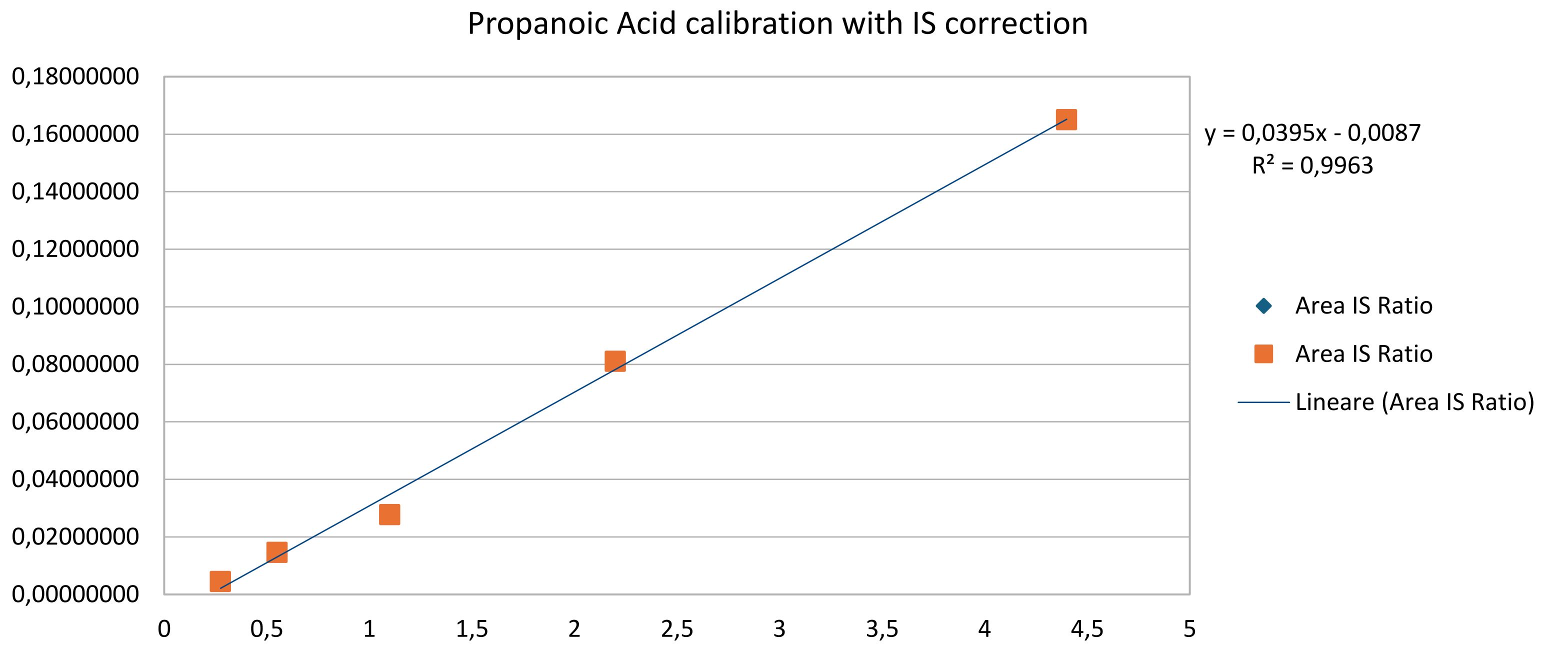

LOD/LOQ		
Slope =	4787,8	
σ cal. Curve =	563,6	
R2	0,99418	
LOD =	**0,4**	uM
LOQ =	**1,2**	uM

4787,753213	-1059,0227
167,9987652	381,5320015
0,996319823	563,5601151
812,1782096	3
257947802,1	952800,01

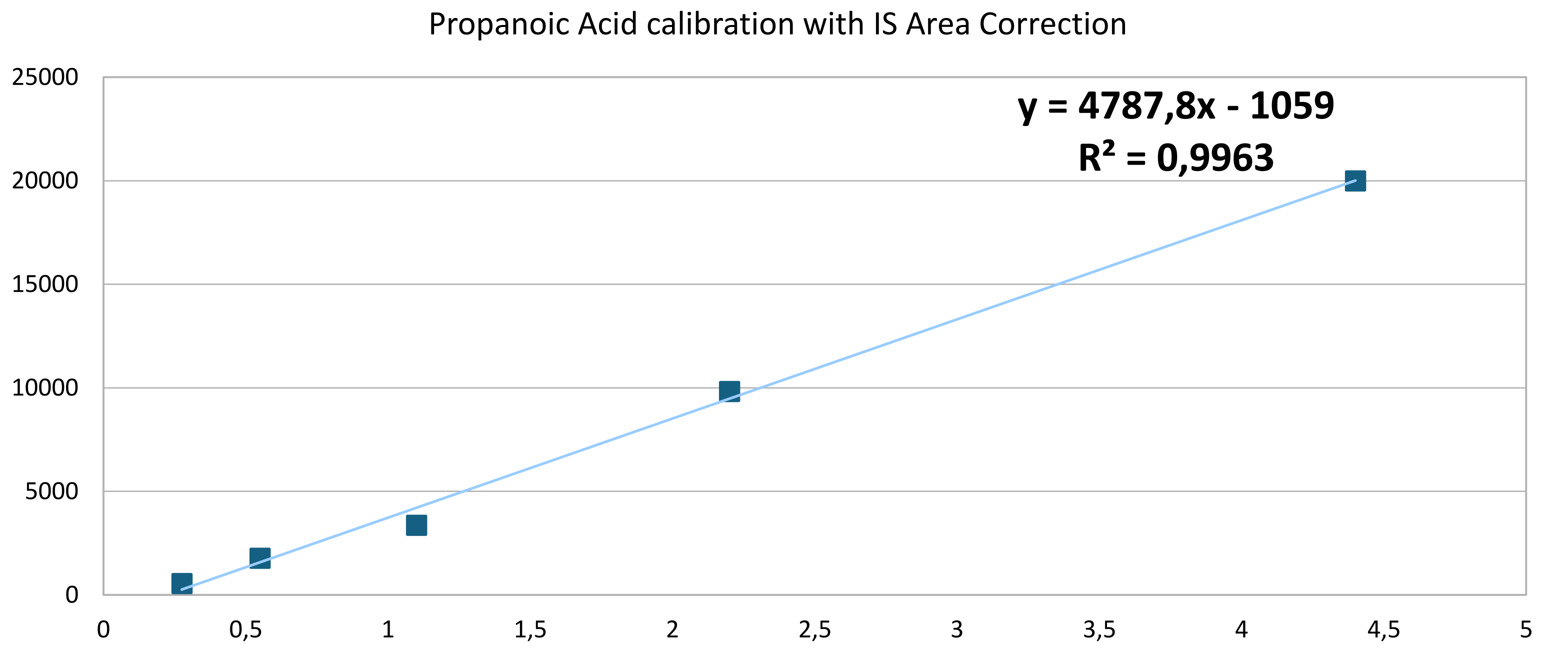

Supplement: Supplementary file 1 [file metabolites-16-00636-s001.zip › Table S10.pdf]
