# Supplementary material for: A Derivatization-Free GC–MS Method for Reliable Quantification of Short-Chain Fatty Acids in Sparus aurata
Source: Metabolites. 2026 Aug 31;16(9):636. doi: 10.3390/metabo16090636 (PMC13609257; doi:10.3390/metabo16090636)

Table S11: pure butyric acid standard calibration and correction with ethyl butyrate as internal standard (IS) for method ExB

Inj. No	Injection Name (Selected Peak)	Type Concentr uM	Ret.Time min Acetic-Butyric Butyric acid	Amount µM Acetic-Butyric Butyric acid	Rel.Area % Acetic-Butyric Butyric acid	Area counts*min Acetic-Butyric **Area blanked**	IS RATIO **Area IS Ratio**	RAW Area	BLANK REF AREA 0 **Corrected Sample Area**
105	STDs point 1	**10,8**	7,669	n.a.	22,26	**72779,528**	**0,60088571**	72779,5275	**72779,5275**
107	STDs point 2	**5,4**	7,685	n.a.	21,57	**26468,114**	**0,25679913**	26468,1139	**31103,61728**
109	STDs point 3	**2,7**	7,669	n.a.	20,47	**12487,225**	**0,12736586**	12487,2254	**15426,60579**
111	STDs point 4	**1,35**	7,669	n.a.	16,59	**6450,8040**	**0,066731783**	6450,804	**8082,581423**
113	STDs point 5	**0,675**	7,669	n.a.	11,16	**3135,1301**	**0,032706703**	3135,1301	**3961,449569**

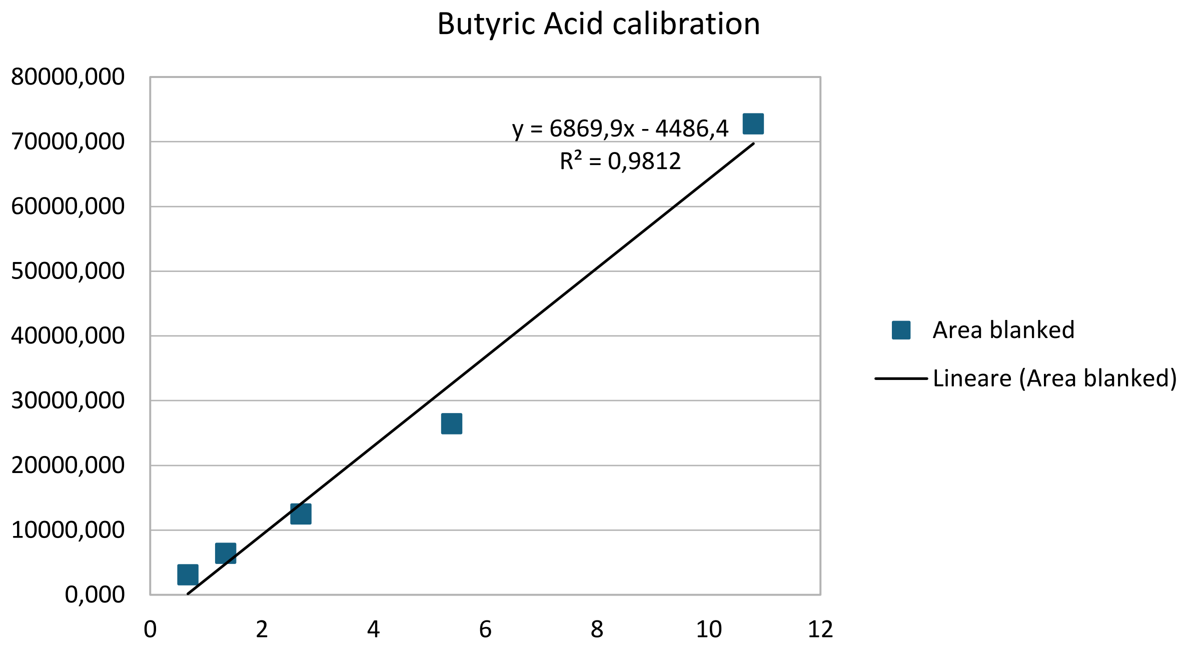

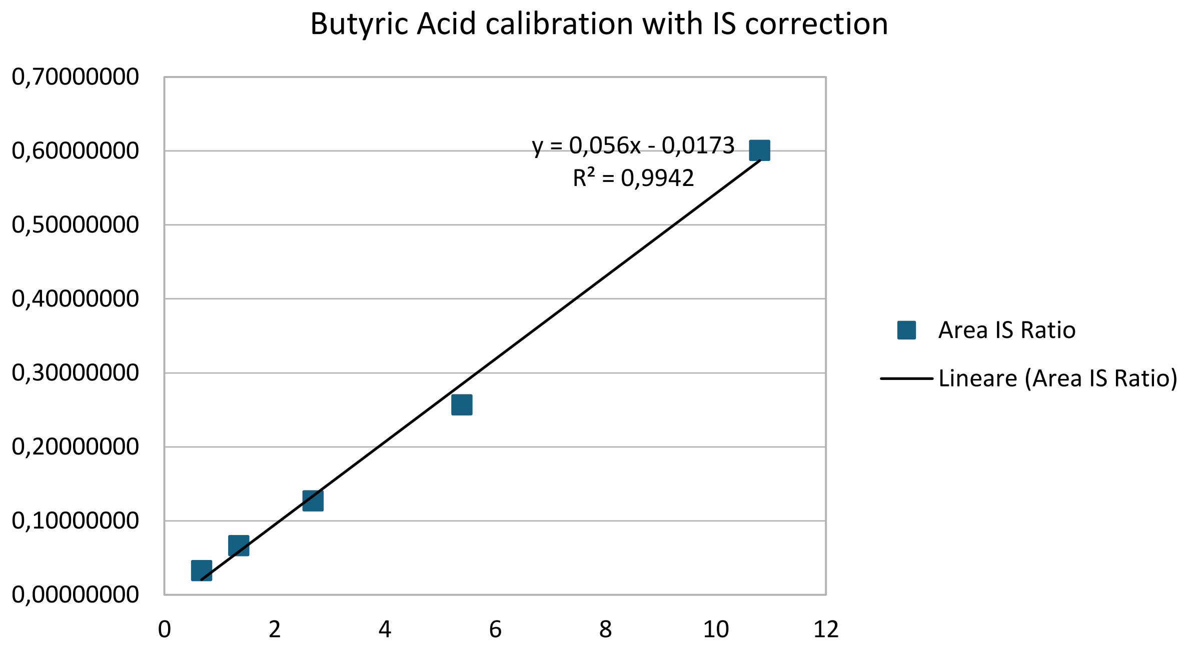

LOD/LOQ		
Slope =	6777,6	
σ cal. Curve =	2465,1	
R2	0,99418	
LOD =	**1,2**	uM
LOQ =	**3,6**	uM

6777,588293	-2093,450692
299,3828632	1668,871498
0,994180442	2465,086572
512,5030955	3
3114302861	18229955,42

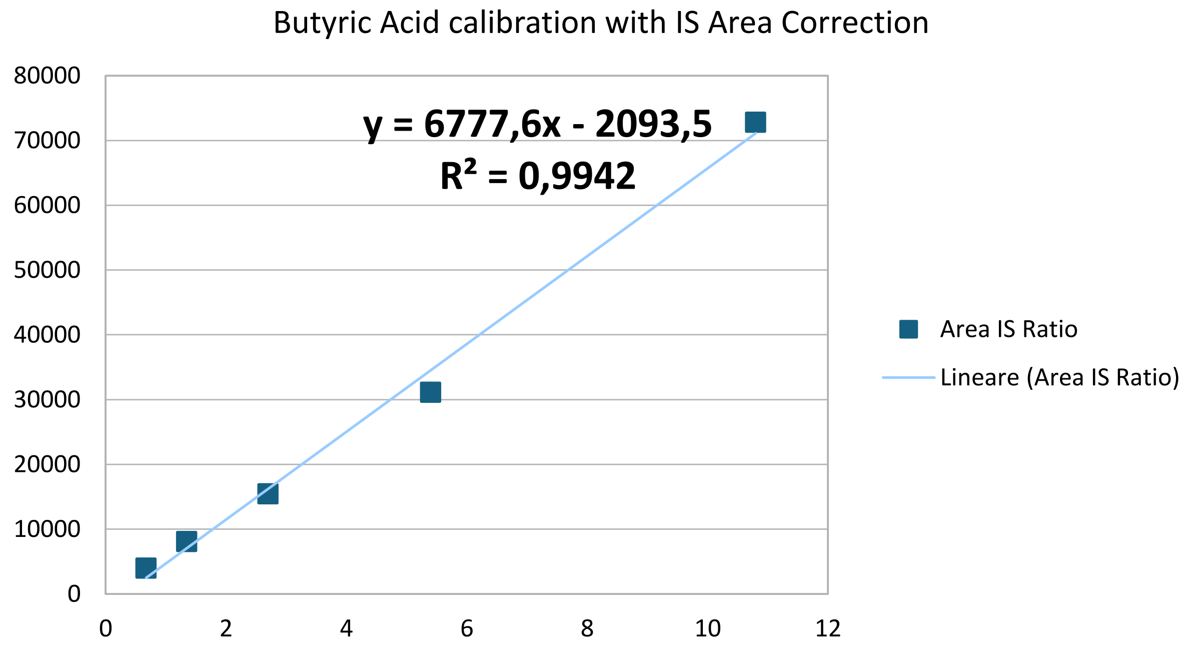

Supplement: Supplementary file 1 [file metabolites-16-00636-s001.zip › Table S11.pdf]
