# Supplementary material for: A Derivatization-Free GC–MS Method for Reliable Quantification of Short-Chain Fatty Acids in Sparus aurata
Source: Metabolites. 2026 Aug 31;16(9):636. doi: 10.3390/metabo16090636 (PMC13609257; doi:10.3390/metabo16090636)
Supplement: Supplementary file 1 [file metabolites-16-00636-s001.zip › Table S12.pdf]

Table S12: ethylbutyrate alone used as internal standard (IS) for injection bias correction in method ExB

Inj.	Injection Name	Type	Ret.Time	Amount	Rel.Area	Area	Sample Area Correction
No	(Selected Peak)		min	n.a.	%	counts*min	
			IS_ethylbutyric	IS_ethylbutyric	IS_ethylbutyric	IS_ethylbutyric	
105	STDs point 1	Unknown	4,319	n.a.	81,51	121120,4175	1
107	STDs point 2	Unknown	4,318	n.a.	82,64	103069,3304	1,175135387
109	STDs point 3	Unknown	4,319	n.a.	88,53	98042,173	1,235390993
111	STDs point 4	Unknown	4,319	n.a.	85,18	96667,6403	1,252957216
113	STDs point 5	Unknown	4,318	n.a.	89,52	95855,8881	1,26356784
						MAX	
						121120,4175	
