# Supplementary material for: A Derivatization-Free GC–MS Method for Reliable Quantification of Short-Chain Fatty Acids in Sparus aurata
Source: Metabolites. 2026 Aug 31;16(9):636. doi: 10.3390/metabo16090636 (PMC13609257; doi:10.3390/metabo16090636)
Supplement: Supplementary file 1 [file metabolites-16-00636-s001.zip › Table S13.pdf]

Table S13: Volumes of recovered organic phase and percentage of recovery per each sample, extracted with either the ExA or ExB method.

	ExA								ExB		
	Volume of recovered MTBE (µL)				Fraction of recovered MTBE (%)					Volume of recovered MTBE (µL)	Fraction of recovered MTBE (%)
Sample	1°	2°	3°	Total	1°	2°	3°	Total	Sample		
1	380	0	148	528	38,00%	0,00%	74,00%	37,71%	1	800	80,00%
2	660	0	0	660	66,00%	0,00%	0,00%	47,14%	2	800	80,00%
3	100	60	210	370	10,00%	30,00%	105,00%	26,43%	3	900	90,00%
4	210	100	30	340	21,00%	50,00%	15,00%	24,29%	4	900	90,00%
5	480	20	300	800	48,00%	10,00%	150,00%	57,14%	5	800	80,00%
6	171	118	190	479	17,10%	59,00%	95,00%	34,21%	6	900	90,00%
7	110	180	310	600	11,00%	90,00%	155,00%	42,86%	7	850	85,00%
8	650	130	30	818	65,00%	65,00%	15,00%	58,43%	8	900	90,00%
9	110	115	127	352	11,00%	57,50%	63,50%	25,14%	9	800	80,00%
10	110	190	100	400	11,00%	95,00%	50,00%	28,57%	10	900	90,00%
11	400	110	290	800	40,00%	55,00%	145,00%	57,14%	11	800	80,00%
12	330	150	30	510	33,00%	75,00%	15,00%	36,43%	12	800	80,00%
13	183	146	400	729	18,30%	73,00%	200,00%	52,07%	13	900	90,00%
14	200	23	200	423	20,00%	11,50%	100,00%	30,21%	14	800	80,00%
15	339	0	190	529	33,90%	0,00%	95,00%	37,79%	15	850	85,00%
16	200	113	200	513	20,00%	56,50%	100,00%	36,64%	16	850	85,00%
17	66	191	125	382	6,60%	95,50%	62,50%	27,29%	17	900	90,00%
18	210	26	190	426	21,00%	13,00%	95,00%	30,43%	18	900	90,00%
19	730	0	0	730	73,00%	0,00%	0,00%	52,14%	Avg	853	85,28%
20	115	350	100	565	11,50%	175,00%	50,00%	40,36%	SD	47	4,69%
21	400	130	300	830	40,00%	65,00%	150,00%	59,29%	CV	5,5%	5,5%
Avg	293	102	165	561	29,30%	51,24%	82,62%	40,08%			
SD	200	87	113	167	20,01%	43,56%	56,52%	11,94%			
CV	68,3%	85,0%	68,4%	29,8%	68,3%	85,0%	68,4%	29,79%			

	Recovered MTBE volumes (µL)			CV
ExA 1°	293	±	200	68,3%
ExA 2°	102	±	87	85,0%
ExA 3°	165	±	113	68,4%
ExA total	561	±	167	29,8%
ExB total	853	±	47	5,5%

	Recovered MTBE fractions (%)			CV
ExA 1°	29,3%	±	20,0%	68,3%
ExA 2°	51,2%	±	43,6%	85,0%
ExA 3°	82,6%	±	56,5%	68,4%
ExA total	40,1%	±	11,9%	29,79%
ExB total	85,3%	±	4,7%	5,5%
