# Supplementary material for: A Derivatization-Free GC–MS Method for Reliable Quantification of Short-Chain Fatty Acids in Sparus aurata
Source: Metabolites. 2026 Aug 31;16(9):636. doi: 10.3390/metabo16090636 (PMC13609257; doi:10.3390/metabo16090636)

Table S2: calibration in matrix for propionic acid, method ExA

No	Injection Name		Ret.Time min siim60	Area counts*min siim60	Height counts siim60	Amount n.a. siim60	Rel.Area % siim60	Peak Type siim60	Area normalized	Height normalized
26	Pure_STD_7.5	0,15	6,72	2825247,1	112546647	n.a.	21,22	BMB	0,102426	0,16485
22	Pure_STD_15	0,3	6,721	4900932,7	212851693	n.a.	99,19	BMB	0,187185	0,320586
23	Pure_STD_30	0,6	6,72	9834276,3	425446686	n.a.	93,22	BMB	0,374875	0,642839
18	23-10_mix_5uL	0,3	6,736	977121,83	31474679,1	n.a.	20,46	BMB*	0,402742	0,54752
14	23-10_mix_10uL	0,61	6,72	2855081,4	139710447	n.a.	23,76	BMB	1,034611	1,937486
10	23-10_mix_15uL	0,91	6,72	3362118,9	147451518	n.a.	24,81	BMB	1,360592	2,394591
6	23-10_mix_30uL	1,82	6,72	4918795,8	222593662	n.a.	26,25	BMB	2,757354	4,871511
2	23-10_mix_0uL	*0,01087*	6,72	16551,878	600706,486	n.a.	5,26	MB	0,005148	0,007319

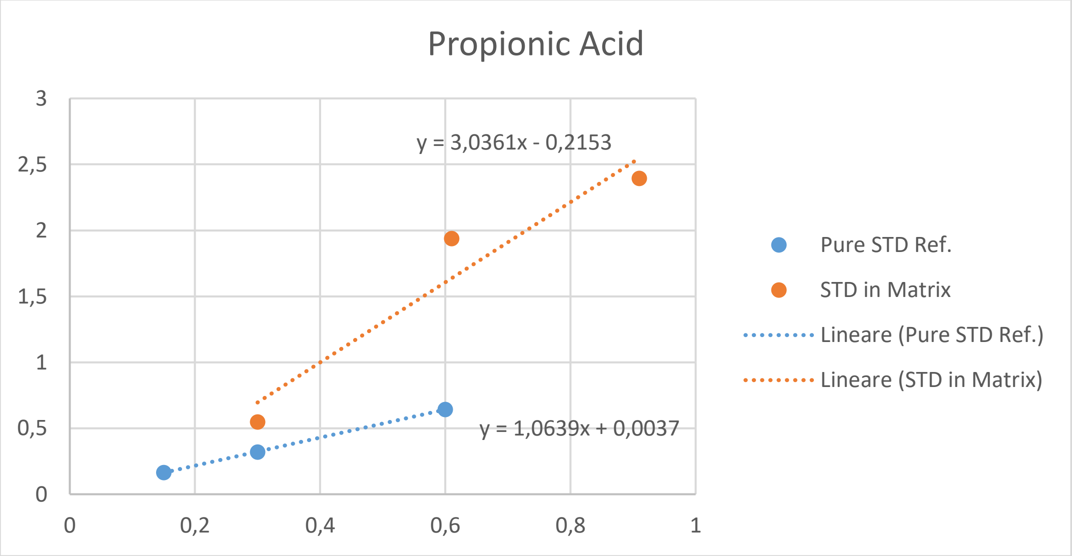

Supplement: Supplementary file 1 [file metabolites-16-00636-s001.zip › Table S2.pdf]
