# Supplementary material for: A Derivatization-Free GC–MS Method for Reliable Quantification of Short-Chain Fatty Acids in Sparus aurata
Source: Metabolites. 2026 Aug 31;16(9):636. doi: 10.3390/metabo16090636 (PMC13609257; doi:10.3390/metabo16090636)

Table S3: calibration in matrix for butyric acid, method ExA

No	Injection Name		Ret.Time min siim60	Area counts*min siim60	Height counts siim60	Amount blaked height	Rel.Area % siim60	Peak Type siim60	Area normalized	Height normalized
26	Pure_STD_7.5_0.15mM	0,15	7,703	10414760,7	351036995,6	351036995,6	78,22	BMB	0,377575	0,514173
22	Pure_STD_15_0.3mM	0,3	7,687	14792541,9	463061904,6	463061904,6	74,96	BMB*	0,564982	0,69744
23	Pure_STD_30_0.6mM	0,6	7,689	29641279,8	924412880,3	926855983,5	74,96	BMB*	1,129901	1,396765
18	23-10_mix_5uL_0.3mM	0,3	7,703	3766975,3	182027786,4	177057507,7	78,86	BMB	1,552642	3,080019
14	23-10_mix_10uL_0.61 mM	0,61	7,686	9101216,69	305900561	300930282,3	75,73	BMB	3,298056	4,17326
10	23-10_mix_15uL_0.91mM	0,91	7,702	10131158,8	335269651,2	330299372,5	74,75	BMB	4,099906	5,364013
6	23-10_mix_30uL_1.82mM	1,82	7,686	13754804,4	461294903,8	456324625,1	73,4	BMB	7,7106	9,986765
2	23-10_mix_0uL	*0,01087*	7,702	161509,204	4970278,68	**4970278,68**	33,93	BMB*	0,050236	0,06056

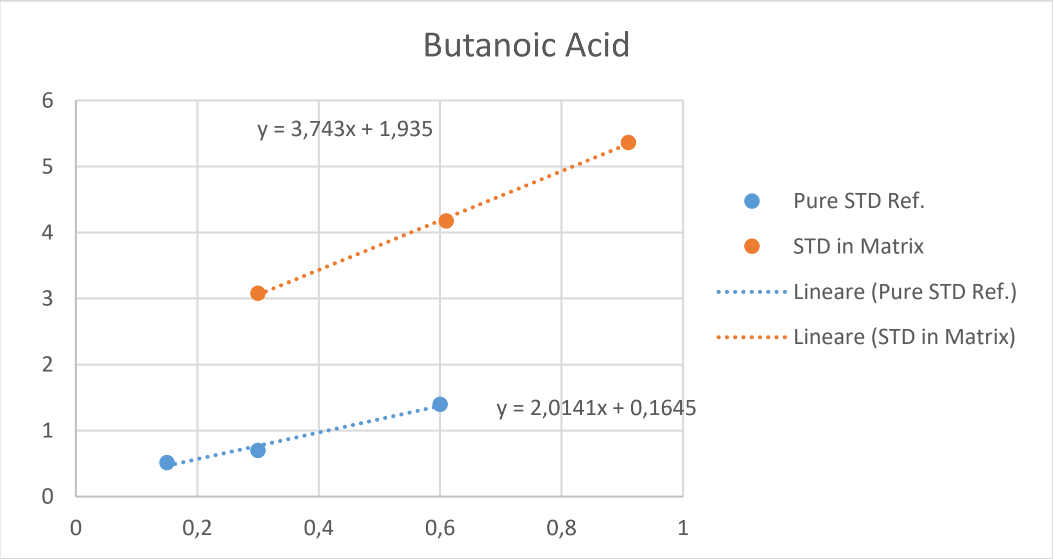

Supplement: Supplementary file 1 [file metabolites-16-00636-s001.zip › Table S3.pdf]
