# Supplementary material for: A Derivatization-Free GC–MS Method for Reliable Quantification of Short-Chain Fatty Acids in Sparus aurata
Source: Metabolites. 2026 Aug 31;16(9):636. doi: 10.3390/metabo16090636 (PMC13609257; doi:10.3390/metabo16090636)
Supplement: Supplementary file 1 [file metabolites-16-00636-s001.zip › Table S4.pdf]

Table S4: biphenyl injection bias correction in matrix, method ExA

No	Injection Name	Ret.Time min TIC_F1	Area counts*min TIC_F1	Height counts TIC_F1	Amount n.a. TIC_F1	Rel.Area % TIC_F1	Peak Type TIC_F1
26	Pure_STD_7.5	11,313	27583259,39	682720933	n.a.	37,09	BM
22	Pure_STD_15	11,313	26182303,62	663945475	n.a.	28,76	BM
23	Pure_STD_30	11,313	26233510,17	661824405	n.a.	24,92	BM
18	23-10_mix_10uL	11,313	2426170,491	57485850,15	n.a.	13,46	BMB
14	23-10_mix_10uL	11,313	2759570,131	72109156,39	n.a.	7,11	BMB
10	23-10_mix_15uL	11,313	2471071,282	61576916,64	n.a.	5,57	BM
6	23-10_mix_30uL	11,313	1783882,524	45692937,57	n.a.	3,12	BMB
2	23-10_mix_0uL	11,313	3214990,435	82072233,08	n.a.	42,35	BM
