# Supplementary material for: A Derivatization-Free GC–MS Method for Reliable Quantification of Short-Chain Fatty Acids in Sparus aurata
Source: Metabolites. 2026 Aug 31;16(9):636. doi: 10.3390/metabo16090636 (PMC13609257; doi:10.3390/metabo16090636)
Supplement: Supplementary file 1 [file metabolites-16-00636-s001.zip › Table S5.pdf]

**Table S5: matrix effect estimation for acetic acid using method ExB**

| Inj. No | Injection Name (Selected Peak) | Type | Ret.Time min Acetic-Butyric Acetic acid | Amount µM Acetic-Butyric Acetic acid | Rel.Area % Acetic-Butyric Acetic acid | Area counts*min Acetic-Butyric Acetic acid | IS Corrected Area | Added Area | Compared to | Ratio Matrix/Pure % |
|---|---|---|---|---|---|---|---|---|---|---|
| 13 | STDs_Matrix P0 | Unknown | 6,703 | n.a. | 41,58 | 88242,8198 | 1,213363561 | 0 | | |
| 15 | STDs_Matrix P1 | Unknown | 6,719 | n.a. | 60,58 | 183584,2183 | 2,377780553 | 95341,3985 | Pure P2 | 1,120118143 |
| 17 | STDs_Matrix P2 | Unknown | 6,719 | n.a. | 54,81 | 127309,7094 | 1,566816249 | 39066,88961 | Pure P3 | 1,115989505 |

**Matrix effect**
**11,8%**
