# Supplementary material for: A Derivatization-Free GC–MS Method for Reliable Quantification of Short-Chain Fatty Acids in Sparus aurata
Source: Metabolites. 2026 Aug 31;16(9):636. doi: 10.3390/metabo16090636 (PMC13609257; doi:10.3390/metabo16090636)
Supplement: Supplementary file 1 [file metabolites-16-00636-s001.zip › Table S6.pdf]

Table S6: matrix effect estimation for propionic acid using method ExB

Inj. No	Injection Name (Selected Peak)	Type	Ret.Time min Propanoic Propanoic acic	Amount µM Propanoic Propanoic acic	Rel.Area % Propanoic Propanoic acic	Area counts*min Propanoic Propanoic acic	IS Corrected Area	Added Area	Compared to	Ratio Matrix/Pure %
13	STDs_Matrix P0	Unknown	7,163	n.a.	3,42	12984,4519	0,178539861	0		
15	STDs_Matrix P1	Unknown	7,163	n.a.	100	22146,0105	0,286834857	9161,5586	Pure P2	1,00786374
17	STDs_Matrix P2	Unknown	7,163	n.a.	100	16746,2575	0,20609825	3761,8056	Pure P3	1,088064484

Matrix effect
4,8%
