# Supplementary material for: A Derivatization-Free GC–MS Method for Reliable Quantification of Short-Chain Fatty Acids in Sparus aurata
Source: Metabolites. 2026 Aug 31;16(9):636. doi: 10.3390/metabo16090636 (PMC13609257; doi:10.3390/metabo16090636)
Supplement: Supplementary file 1 [file metabolites-16-00636-s001.zip › Table S7.pdf]

Table S7: matrix effect estimation for butyric acid using method ExB

Inj. No	Injection Name (Selected Peak)	Type	Ret.Time min Acetic-Butyric Butyric acid	Amount µM Acetic-Butyric Butyric acid	Rel.Area % Acetic-Butyric Butyric acid	Area counts*min Acetic-Butyric Butyric acid	IS Corrected Area	Added Area	Compared to	Ratio Matrix/Pure %
13	STDs_Matrix P0	Unknown	7,669	n.a.	34,77	67935,112	0,934126874	0		
15	STDs_Matrix P1	Unknown	7,669	n.a.	29,49	97899,2266	1,267989587	29964,1146	Pure P2	1,132083484
17	STDs_Matrix P2	Unknown	7,669	n.a.	31,64	83109,3316	1,022836764	15174,2196	Pure P3	1,215179443

Matrix effect
17,4%
