# Supplementary material for: A Derivatization-Free GC–MS Method for Reliable Quantification of Short-Chain Fatty Acids in Sparus aurata
Source: Metabolites. 2026 Aug 31;16(9):636. doi: 10.3390/metabo16090636 (PMC13609257; doi:10.3390/metabo16090636)
Supplement: Supplementary file 1 [file metabolites-16-00636-s001.zip › Table S8.pdf]

Table S8: ethyl butyrate reference spike-in injections in matrix with method ExB

Inj. No	Injection Name (Selected Peak)	Type	Ret.Time min IS_ethylbutyric	Amount n.a. IS_ethylbutyric	Rel.Area % IS_ethylbutyric	Area counts*min IS_ethylbutyric
13	STDs_Matrix P0	Unknown	4,318	n.a.	24,08	72725,7869
15	STDs_Matrix P1	Unknown	4,318	n.a.	20,55	77208,226
17	STDs_Matrix P2	Unknown	4,319	n.a.	22,58	81253,7587
