# Supplementary material for: A Derivatization-Free GC–MS Method for Reliable Quantification of Short-Chain Fatty Acids in Sparus aurata
Source: Metabolites. 2026 Aug 31;16(9):636. doi: 10.3390/metabo16090636 (PMC13609257; doi:10.3390/metabo16090636)

Table S9: pure acetic acid standard calibration and correction with ethyl butyrate as internal standard (IS) for method ExB

Inj. No	Injection Name (Selected Peak)	Type<br>**Concentr uM**	Ret.Time min<br>Acetic-Butyric<br>Acetic acid	Amount µM<br>Acetic-Butyric<br>Acetic acid	Rel.Area %<br>Acetic-Butyric<br>Acetic acid	Area counts*min<br>Acetic-Butyric<br>**Area blanked**	IS RATIO<br>**Area IS Ratio**	RAW Area	BLANK REF AREA<br>3404<br>**Corrected Sample Area**
105	STDs point 1	**87,1**	6,703	n.a.	70,93	**228552,57**	**1,8869863**	231956,5742	**228552,5742**
107	STDs point 2	**43,55**	6,719	n.a.	66,02	**81713,270**	**0,79279908**	85117,2701	**96024,15531**
109	STDs point 3	**21,775**	6,719	n.a.	57,39	**31602,503**	**0,32233580**	35006,5027	**39041,44721**
111	STDs point 4	**10,8875**	6,702	n.a.	48,03	**15269,304**	**0,15795673**	18673,3044	**19131,78514**
113	STDs point 5	**5,44375**	6,719	n.a.	39,99	**7829,9897**	**0,081685015**	11233,9897	**9893,723174**

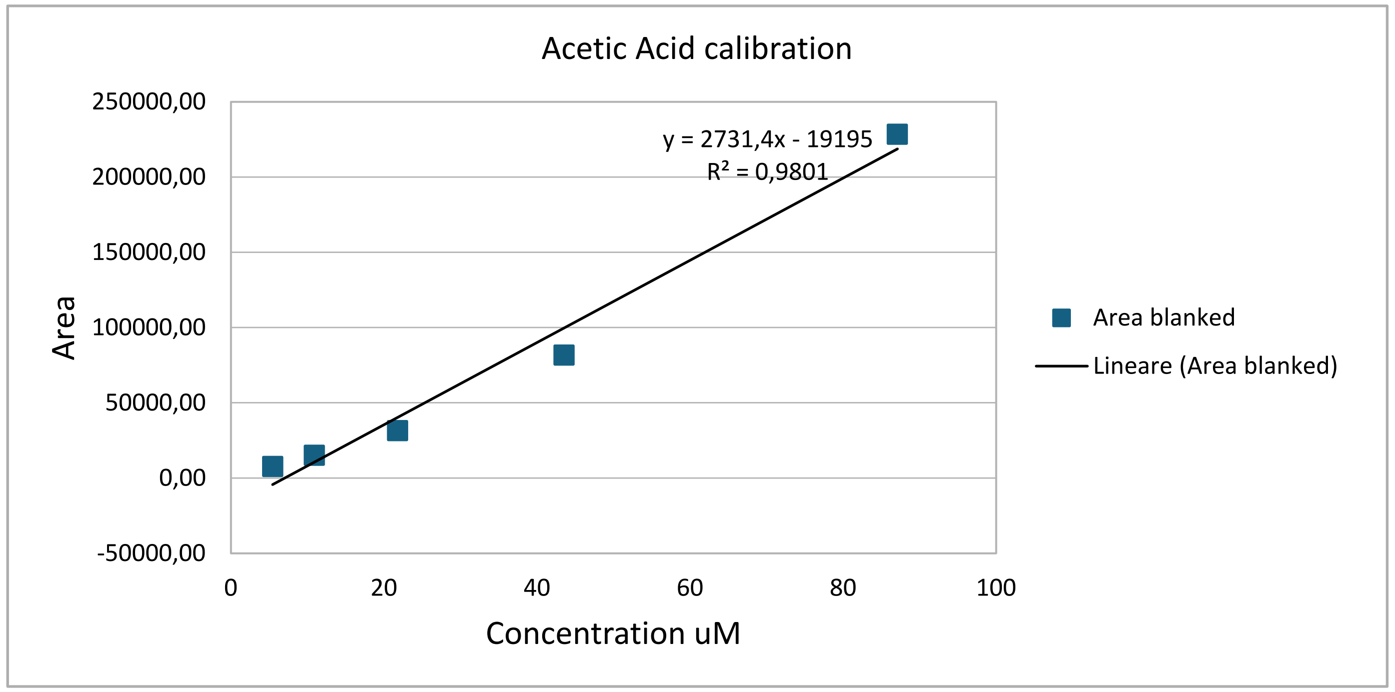

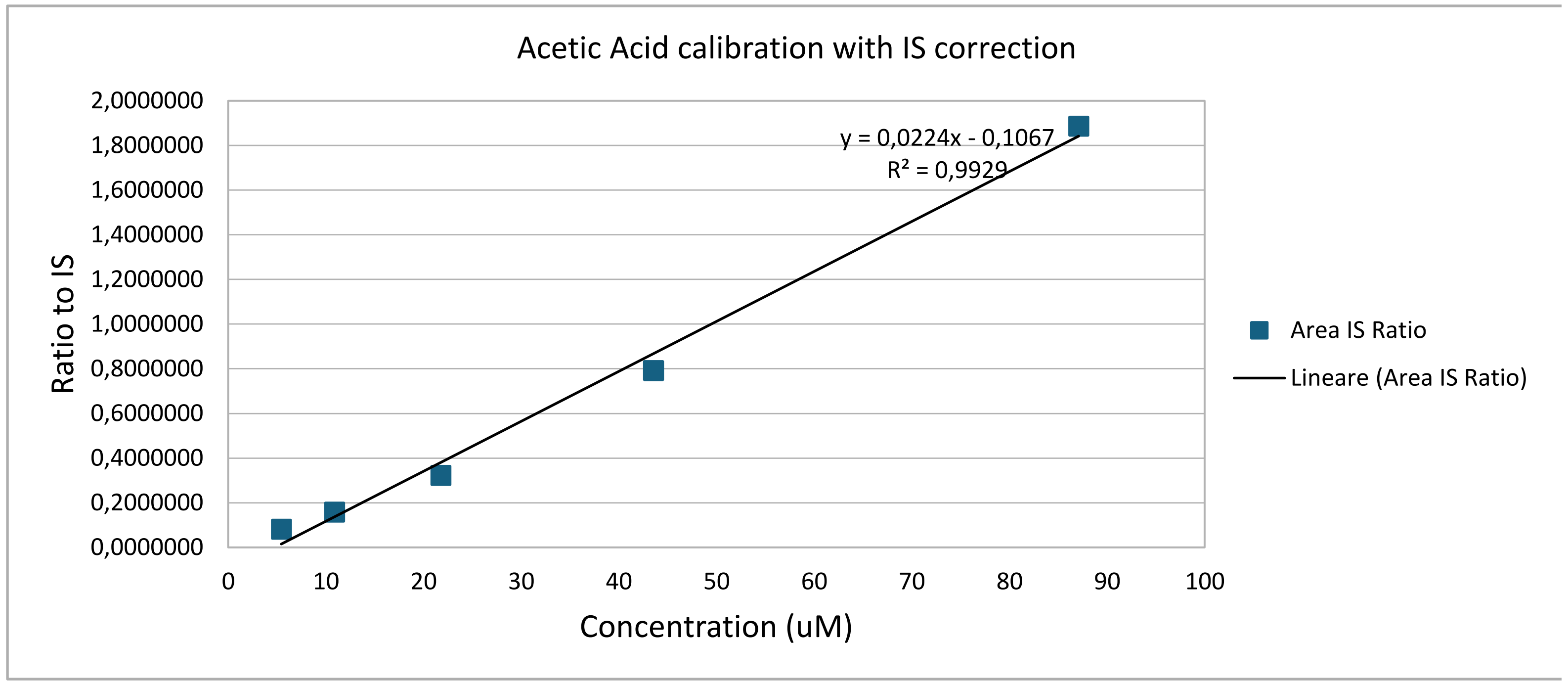

LOD/LOQ		
Slope =	2709,7	
σ cal. Curve =	8813,5	
R2	0,99418	
LOD =	**10,7**	uM
LOQ =	**32,5**	uM

2709,735028	-12928,2074
132,7238783	5966,771731
0,992854194	8813,505944
416,8266934	3
32378216798	233033661,1

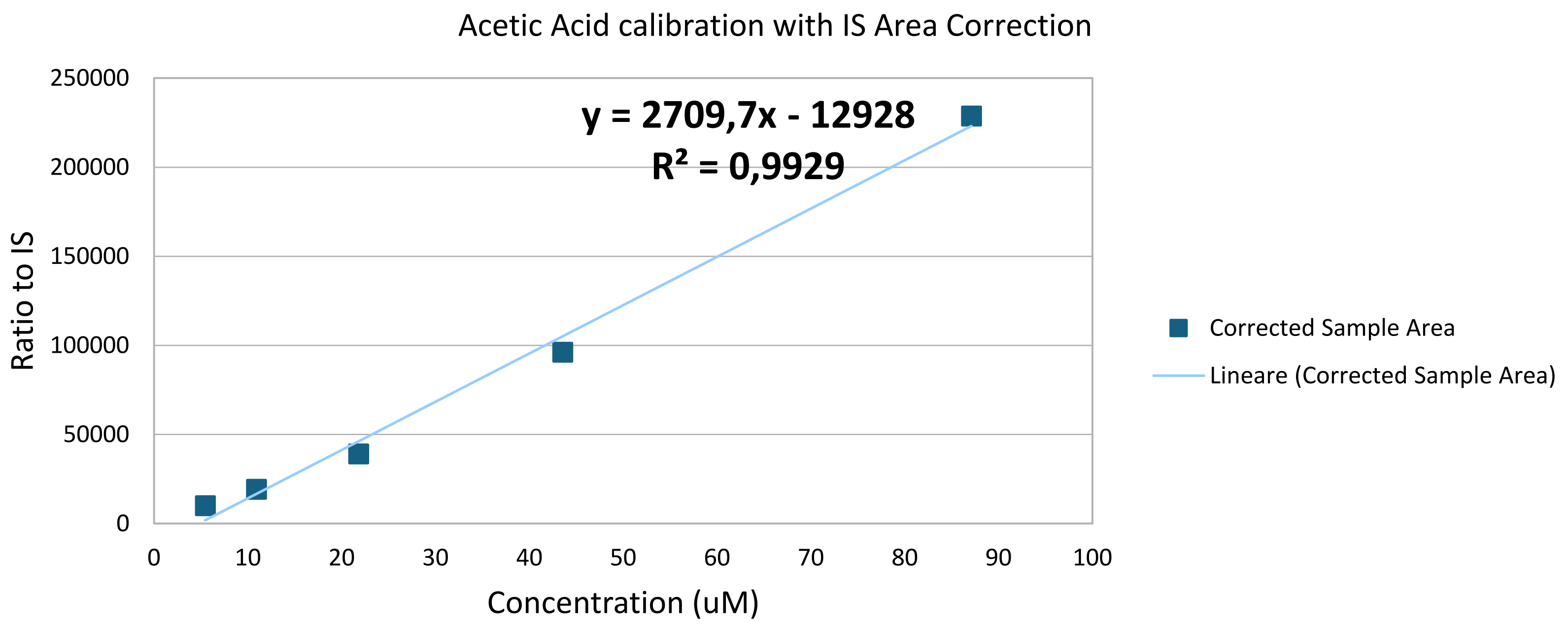

Supplement: Supplementary file 1 [file metabolites-16-00636-s001.zip › Table S9.pdf]
